# Analysis of the Matrix Metalloproteinases Family Profile in Gastric Cancer Suggests Key Matrix Metalloproteinases for Tumor Development and Their Clinical Impact

**DOI:** 10.1002/mc.70097

**Published:** 2026-02-23

**Authors:** Aline Costa Bastos, André Salim Khayat, Emanuele Raimunda Louzada Moraes, Ágatha Tereza Miranda Tavares, Ronald Matheus da Silva Mourão, Fabiano Cordeiro Moreira, Samir Mansour Moraes Casseb, Samia Demachki, Geraldo Ishak, Williams Fernandes Barra, Rommel Mario Rodríguez Burbano, Paulo Pimentel de Assumpção

**Affiliations:** ^1^ Oncology Research Center Federal University of Pará Belem Pará Brazil; ^2^ Institute of Biological Sciences Federal University of Pará Belem Pará Brazil; ^3^ Molecular Biology Laboratory Hospital Ophir Loyola Belém Pará Brazil

**Keywords:** extracellular matrix, matrix metalloproteinases, sequence analysis, RNA

## Abstract

Gastric cancer (GC) is the fifth most common type worldwide, representing a public health problem. Among the genes related to this tumorigenesis, the family of matrix metalloproteinases (MMPs), essential regulators of the extracellular matrix (ECM), stand out for their involvement in the development and progression of GC. Therefore, we aimed to evaluate *MMP* gene expression variation, its relationship with clinicopathological factors and its transcriptome‐wide associations. To this end, RNAseq, correlation network, and biological pathway enrichment analyses were performed on tumor samples from GC and peritumoral samples from patients treated at a reference center in the Northern region of Brazil. Among the 22 investigated *MMPs*, seven genes (*MMP2, MMP3, MMP10, MMP12, MMP14, MMP15*, and *MMP16*) were upregulated in cancer, while *MMP8* was downregulated. Increased expression of seven of the eight differentially expressed *MMPs* was found in early stages of the disease compared to Tumor, Node, Metastasis (TNM) stage IV. *MMP16* showed higher expression in the diffuse‐type gastric adenocarcinoma. An increased expression of *MMP10* was observed in the EBV/TCGA group. A significant reduction in survival was noticed in those patients with lower expression of *MMP8, MMP12*, and *MMP14*. Transcriptomic correlation analyses demonstrated that differentially expressed *MMPs* interact with genes likely involved in cell adhesion, ECM organization, and immune response, such as *COL1A2, CDH11, KIRREL1, PPP1R14D, CEACAM8, ZNF423*, and *PRRX1*. The enrichment of biological pathways suggests involvement in processes such as ECM organization, collagen and proteoglycan degradation, suggesting that these genes possibly are involved in carcinogenic dynamics, supporting the role of *MMPs* in tumor ECM reorganization.

**Abbreviations**
AGS: gastric adenocarcinoma
AJCC: American Joint Committee on Cancer
CIN: Chromosomal Instability
CPM: Constant Potts Model
cTNM: TNM classification
DGE: differentially expressed gene
EBV: Epstein‐Barr virus
ECM: extracellular matrix
FLOT: 5‐Fluorouracil, Leucovorin, Oxaliplatin, and Taxane
GAC: gastric adenocarcinoma
GC: gastric cancer
GS: genomically stable
HUJBB: João de Barros Barreto University Hospital
*H. pylori*
: *Helicobacter pylori*
miRNAs: microRNAs
*MMP*
: matrix metalloproteinase
MSI: Microsatellite Instability
NES: Normalized Enrichment Score
RIN: RNA integrity number
PTT: peritumoral tissue
T: tumor
TCGA: The Cancer Genome Atlas Program
TCGA‐STAD: The Cancer Genome Atlas Stomach Adenocarcinoma
*TIMP*
: tissue inhibitors
TNM: Tumor, Node, Metastasis
UFPA: federal University of Pará
ypN: pathological staging of lymph nodes

## 1 Introduction

The gastric cancer (GC) ranks fifth among cancers with the highest mortality rates and fifth in terms of incidence worldwide in 2022 [1]. This malignant neoplasm has several classifications, with adenocarcinoma accounting for 95% of GC cases. It can be differentiated by its anatomical location, distinguishing between cases in the cardia and non‐cardia regions; and by the histology of the cells, also known as the Lauren classification, which divides cases into two categories: the diffuse type, associated with hereditary genetic abnormalities, and the intestinal type, related to environmental factors such as smoking, high salt intake, and infection by *Helicobacter pylori* (*H. pylori*), which is the most frequent type [2, 3].

Although various therapeutic alternatives are available, and even with established chemotherapy protocols, the choice of therapy is influenced by the type of cancer and the patient's individuality. In this regard, it's important to note that most tumors exhibit somatic mutations, characterizing genomic heterogeneity and conferring survival advantages to the malignant neoplasm [4, 5, 6]. Therefore, one promising alternative to overcome current therapeutic challenges is the detailed molecular study of GC, aiming to identify modifications in the structure or expression of genes that may contribute to the formation or progression of cancer [7].

One of the gene families associated with the tumorigenesis of GC is the matrix metalloproteinases. MMP proteins belong to the family of zinc‐dependent endopeptidases and are physiologically secreted into the pericellular space, in the ECM, and can also be found on the cell surface [8].

This opens doors to various functions, such as the regulated degradation of the ECM through the breakdown of collagen, elastin, fibronectin, and proteoglycans, and the regulation of several signaling proteins that mediate intercellular and intracellular communication, regulation of cytoskeletal proteins, among other activities. These activities, combined with different structural combinations (pro‐peptide and catalytic domains, along with variable domains like hemopexin), classify these enzymes into various subfamilies, such as collagenases (MMP1, MMP8, and MMP13), gelatinases (MMP2 and MMP9), stromelysins (MMP3, MMP10, and MMP11), membrane‐type metalloproteinases (MMP14, MMP15, MMP16, MMP17, and MMP24), etc [9, 10, 11].

Being highly functional enzymes with easy systemic distribution, *MMP* must be meticulously regulated from transcription through post‐translation, involving cell‐to‐cell and cell‐to‐matrix signaling pathways, cytokines, microRNAs (miRNAs), tissue inhibitors (*TIMP*), among other regulation mechanisms [10]. Therefore, *MMP* acts as ECM remodelers, and through their dysregulated proteolytic activity, they contribute to tumor progression [11, 12, 13].

Such alterations inherent to cancer are encompassed within a vast heterogeneity of GC, varying across geographic regions, ethnicity, culture, lifestyle, and ancestry [14, 15, 16, 17, 18]. Due to this, there is a significant difference in GC mortality across different regions in Brazil. These peculiarities also complicate the selection of the best therapeutic approach and management for the patient [14, 15].

The Northern region of Brazil has one of the highest incidence rates of GC in the country [16]. This elevated incidence may be associated with multiple factors, including sociocultural aspects [17, 18]. Many patients do not seek medical care during the early stages of symptom onset due to financial limitations that restrict access to private healthcare services, as well as difficulties related to transportation, food, and lodging for individuals who do not reside in capital cities where cancer treatment centers are located [18, 19]. Additionally, cultural reliance on traditional practices, such as the use of teas and medicinal herbs to alleviate symptoms, contributes to delays in seeking care within the Unified Health System [20, 21]. Furthermore, the population in the Northern region of Brazil maintains the habit of consuming salted meats, a practice that favors the proliferation of microorganisms associated with gastric carcinogenesis. Moreover, the salting process may lead to the formation of nitrogenous compounds, such as nitrosamines, which exhibit well‐established carcinogenic activity [22, 23].

Considering the significant role of *MMP* in tissue organization and their impact on carcinogenesis, and consequently, on the patient's prognosis, we aim to investigate the expression of all genes from the *MMP* family in a group of GC patients from a region with a high incidence of this neoplasm, with the objective of assessing the influence of alterations in these extracellular matrix regulators. Moreover, we will explore the association of fluctuations in the expression of these *MMPs* with various other factors related to carcinogenesis, such as clinicopathological factors, the therapeutic strategy used for patients, the relationship between *MMP* expression and disease classification and staging, as well as correlations with the expression of all other genes in the transcriptome.

## 2 Materials and Methods

### 2.1 Characterization of Samples and Ethical Considerations

For this study, we obtained 72 paired samples of tumor and peritumoral tissue, 84 tumor only, and 114 peritumoral tissue only (non‐tumor), totaling 342 samples from 270 patients diagnosed with gastric adenocarcinoma at João de Barros Barreto University Hospital (HUJBB), Federal University of Pará (UFPA, Belém, PA, Brazil). All participants were informed about the research objectives, and samples were collected only after obtaining written consent through the application of the Free and Informed Consent Form. Approval for sample use and study conduct was granted by the Ethics Committee of HUJBB, with protocol number CAAE 47580121.9.0000.5634. Tumor tissue samples were obtained by excising an approximately 50 µg fragment immediately after gastric resection. The specimens were preserved in RNAlater for transportation, and stored at −80°C in a freezer.

### 2.2 Clinical Characterization of Patients

The medical records and clinical data of the patients were reviewed for the presence of gastric adenocarcinoma, Lauren histological subtype, pathological TNM staging (tumor, node, metastasis), TCGA (The Cancer Genome Atlas Program) classification (Microsatellite Instability [MSI], Genomically Stable [GS], Epstein–Barr Virus positive [EBV], Chromosomal Instability [CIN]), and survival. TNM staging followed the 8th edition of the AJCC (American Joint Committee on Cancer) TNM classification for gastric cancer.

### 2.3 Total RNA Extraction

Initially, tissue samples ranging from 50 to 100 ng were macerated, followed by the addition of 1 mL of TRIZOL® reagent for extraction, preserving RNA integrity and cell lysis. After centrifugation at 13,000 rpm for 10 min at 4°C, RNA was recovered through isopropanol precipitation. The precipitated RNA (total RNA) underwent ethanol washing, was dried at room temperature, and its integrity and concentration were analyzed using Qubit 2.0 Fluorometer (Thermo Fisher Scientific, Invitrogen/Life Technologies, California, USA), and 2200 TapeStation System (Agilent Technologies, California, USA). The ideal criteria for total RNA integrity were met with values between 1.8 and 2.2 (A260/A280 ratio), > 1.8 (A260/A230 ratio), and RIN (RNA integrity number) ≥ 5. Finally, the obtained total RNA was stored at −80°C until further use.

### 2.4 cDNA Library Construction

The construction of libraries utilized the TruSeq Stranded Total RNA Library Prep Kit with Ribo‐Zero Gold (Illumina, Inc., California, USA) following the manufacturer's instructions. For library preparation, 1 μg of total RNA per sample was used in a total volume of 10 μL. After library construction, a further integrity assessment was conducted using the 2200 TapeStation System (Agilent Technologies, California, USA).

### 2.5 NGS Sequencing

The previously constructed cDNA libraries were loaded onto the Illumina NextSeq sequencing system and subjected to paired‐end sequencing, where reads were generated from both the positive and negative strands of the cDNA molecules. Sequencing was performed using the NextSeq® 500 ID Output V2 kit—150 cycles (Illumina, Inc., California, USA), following the manufacturer's specified conditions for library processing.

### 2.6 Read Quality Control and Alignment

The quality assessment of the file readings was conducted using FastQC version 0.11.9. Adapters and low‐quality readings were removed using Trimommatic [24]. Subsequently, the filtered and trimmed readings from the previous stage were aligned against the sequences of the human transcriptome, using the coding transcripts in hg v38 as the reference index (available at www.ensembl.org), and quantified using Salmon version 1.5.2 [25]. In R software version 4.1.0 [26], the readings were imported from Salmon using the Tximport package version 3.14.0 [27].

RNA‐seq quantification was performed using Salmon, and transcript abundances were subsequently aggregated to the gene level prior to downstream analysis. DESeq. 2 package version 3.14 [28] was applied using non‐scaled abundance counts, with standard normalization by size factors and correction for multiple tests (Benjamini–Hochberg).

Although the analyses were not paired, the statistical model included covariates to control for differences between sequencing batches and tissue types, thereby reducing technical variability and inter‐dataset effects.

### 2.7 Survival Analysis

After obtaining clinical data from the 77 patients enrolled in the study, those who experienced mortality within 30 days post‐surgery (*n* = 5) were excluded to mitigate the influence of confounding factors on survival outcomes, resulting in a final cohort of 72 patients. Additionally, a cohort of 203 patients from The Cancer Genome Atlas Stomach Adenocarcinoma (TCGA‐STAD) database was included in the analysis. Gene hub expressions were dichotomized into high and low expression levels using the Survminer package version 0.4.9 [29]. Kaplan‐Meier curves were generated using the same package. Significant differences between survival curves (*p* < 0.05) were assessed using the log‐rank test implemented in the Survival package version 3.2‐13 [30].

### 2.8 Correlation Network

Based on the variance‐stabilized transformed expression matrix, we defined as target genes the members of the *MMP* family previously identified as differentially expressed between Tumor and Peritumoral tissues. For each *MMP*, we computed the Spearman correlation (*ρ*) between its expression and that of all other genes, and selected the five partners with the highest |*ρ*| per target. This step focuses the analysis on the most informative “neighborhood” of each *MMP* reducing noise and multiplicity and yields an interpretable subnetwork. Subsequently, we constructed an undirected graph weighted by |*ρ*| , preserving the correlation sign as an edge attribute, and filtered connections with |ρ| ≥ 0.30 to retain only stronger associations. Cluster detection was performed using the Leiden algorithm under the Constant Potts Model (CPM). This choice emphasizes macro‐modules of co‐expression centered around the *MMPs*, which are useful for generating hypotheses of co‐regulation and shared pathways. The visualization layout was obtained using *layout_with_graphopt* from the igraph package version 1.3.5. Networks were rendered with the ggraph package version 2.2.2. Additionally, a heatmap of the corresponding correlation submatrix was generated.

### 2.9 Biological Pathways Enrichment

For the characterization of biological pathways and their associated functions, 22 *MMPs* genes were submitted to functional enrichment analysis. The analysis was performed using the Reactome tool (https://reactome.org/ ) through ClusterProfiler version 4.3.2. Enrichment analysis was conducted for genes within the 93 modules showing correlations between absolute genetic significance values for traits and Module Membership greater than 0.25. We considered functional enrichment terms with adjusted *p* ≤ 0.05 as significant.

### 2.10 Statistical Analysis

Quantitative data were analyzed using either the Wilcoxon or Kruskal–Wallis test, depending on the number of comparisons. Survival analyses were conducted utilizing the Log‐Rank test. A significance level of *p* < 0.05 was employed for all assessments.

## 3 Results

### 3.1 Expression of *MMP* Family Genes in GC

**Figure 1:**
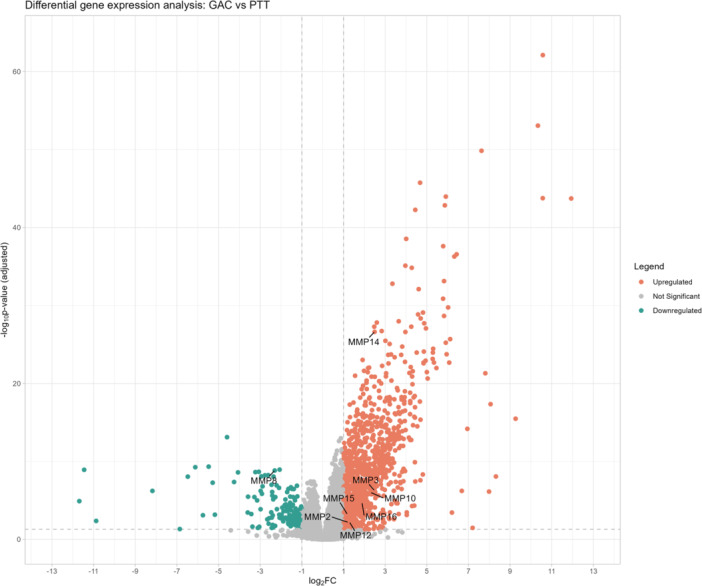
Differential expression of *MMP* family genes. Volcano plot of genes differentially expressed in gastric tumor compared with peritumoral tissue. *MMPs* downregulated (green), *MMPs* with no difference in expression (gray), and *MMPs* upregulated (red), in comparison with expression in peritumoral tissue. With statistical significance of padj < 0.05, |log₂FoldChange| > 1. GAC, gastric adenocarcinoma; PTT, peritumoral tissue.

Samples from tumor and peritumoral tissues from patients diagnosed with GC were analyzed for RNA expression of 22 *MMP* genes (*MMP1, MMP2, MMP3, MMP7, MMP8, MMP9, MMP10, MMP11, MMP12, MMP13, MMP14, MMP15, MMP16, MMP17, MMP19, MMP20, MMP21, MMP24, MMP25, MMP26, MMP27, MMP28*). Among these genes, it was demonstrated that eight *MMP* family genes showed significantly higher expression levels in GC tumor tissues compared to non‐tumor tissues, namely *MMP2* (padj = 6.48 × 10^−3^, |log₂FoldChange| = 1.23), *MMP3* (pad = 5 × 10^−7^, |log₂FoldChange| = 2.48), *MMP10* (padj = 9 × 10^−7^, |log₂FoldChange | = 2.30), *MMP12* (padj = 6 × 10^−3^, |log₂FoldChange| = 1.30), *MMP14* (padj = 2.4 × 10^−27^, |log₂FoldChange| = 2.49), *MMP15* (padj = 5.99 × 10^−4^, |log₂FoldChange| = 1.18), *MMP16* (padj = 1.92 × 10^−5^, |log₂FoldChange| = 1.89). *MMP8* exhibited significantly lower expression levels in tumor tissue compared to non‐tumor tissue (padj = 10^−9^, |log₂FoldChange| = −2.30) (Figure 1 and Table S1).

### 3.2 Relationship Between *MMPs* and Clinicopathological Characteristics

Among the *MMP* that exhibited significant differences in expression, an evaluation was conducted to determine whether these modifications could be associated with the stage of the disease, according to the TNM classification.

**Figure 2:**
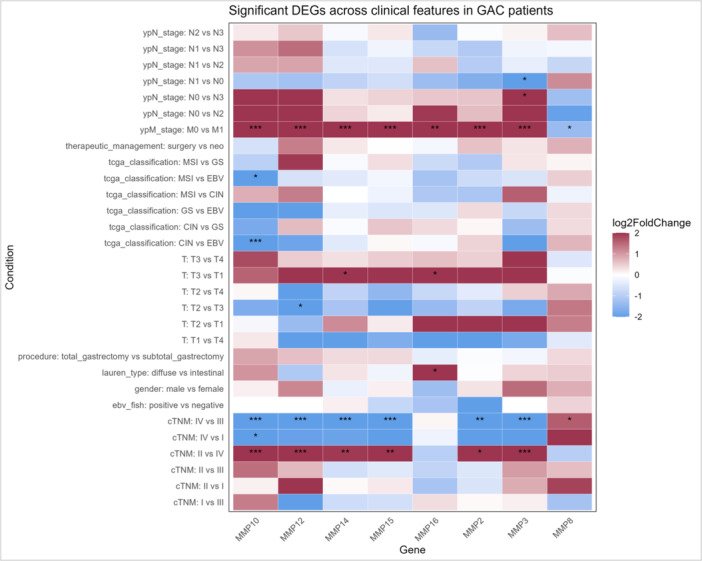
Comparison of *MMP* expression according to clinical characteristics and presentation. Expression levels of the genes *MMP2, MMP3, MMP8, MMP10, MMP12, MMP14, MMP15*, and MMP16 were compared across TNM stages, according to TCGA classification, between diffuse‐type and intestinal‐type tumors, patients who underwent total versus subtotal gastrectomy, male and female patients, and EBV‐positive versus EBV‐negative status. With statistical significance of padj < 0.05, |log₂FoldChange| > 1. CIN, chromosomal instability; cTNM, TNM classification; DGEs, differentially expressed genes; EBV, Epstein–Barr Virus positive; GAC, gastric adenocarcinoma; GS, genomically stable; MSI, microsatellite instability; Neo, neoadjuvant; surgery, adjuvant; T, tumor; ypN, pathological staging of lymph nodes.

When analyzing the expression of *MMPs* (which showed overall significantly higher or lower expression relative to peritumoral tissue) across different TNM stages, we observed increased expression of *MMP2* (padj = 2.1 × 10^−2^), *MMP3* (padj = 9 × 10^−5^), *MMP10* (padj = 2.8 × 10^−6^), *MMP12* (padj = 6.2 × 10^−5^), *MMP14* (padj = 1.4 × 10^−3^), and *MMP15* (padj = 1.2 × 10^−3^) in TNM stage II compared to stage IV. When comparing TNM stage I with stage IV, only *MMP10* exhibited higher expression in stage I. In contrast, when comparing *MMP* expression between TNM stages III and IV, *MMP2* (padj = 2.3 × 10^−3^), *MMP3* (padj = 5.6 × 10^−4^), *MMP10* (padj = 3.6 × 10^−4^), *MMP12* (padj = 2 × 10^−4^), *MMP14* (padj = 4.6 × 10^−6^), and *MMP15* (padj = 4.8 × 10^−5^) were more highly expressed in stage III, whereas only *MMP8* (padj = 4.2 × 10^−2^) showed significantly increased expression in stage IV (Figure 2).

Additionally, the stages of the primary tumor (T), regional lymph nodes (N), and distant metastasis (M) were also analyzed separately according to the TNM classification. *MMP12* (padj = 2.6 × 10^−2^) showed significantly higher expression in patients with T3 tumors when compared to those with T2 tumors. *MMP14* (padj = 4.4 × 10^−2^), and *MMP16* (padj = 4.7 × 10^−2^) showed significantly increased expression in T3 tumors compared to T1 (Figure 2).

*MMPs* gene expression was also analyzed across different N stages. When comparing stage N1 with N0, and N0 with N3, increased expression of *MMP3* (padj = 4 × 10^−2^ for both comparisons) was observed in N0 in both comparisons (Figure 2).

When analyzing *MMP* expression in patients with distant metastasis, *MMP2* (padj = 7.2 × 10^−4^), *MMP3* (padj = 6.4 × 10^−8^), *MMP10* (padj = 3.5 × 10^−11^), *MMP12* (padj = 5.2 × 10^−6^), *MMP14* (padj = 8.3 × 10^−7^), *MMP15* (padj = 6.8 × 10^−6^), and *MMP16* (padj = 3 × 10^−3^) showed higher expression in patients without metastasis (M0) compared to samples from patients with metastasis (M1). However, only *MMP8* (padj = 4.4 × 10^−2^) was found to be significantly expressed in patients M1.

Among the *MMP* that exhibited significant differences in expression, an evaluation was conducted to determine whether these modifications could be associated with specific histological subtypes. Only *MMP16* (padj = 3.4 × 10^‐2^) showed significantly higher expression in diffuse‐type tumors compared to intestinal‐type tumors.

*MMP* expression in gastric cancer was also analyzed according to the TCGA classification. When comparing *MMP* expression between the MSI and EBV groups, as well as between the CIN and EBV groups, an increased expression of *MMP10* (padj = 5.7 × 10^−4^, and padj = 1.9 × 10^−2^, respectively) was observed in the EBV group in both comparisons (Figure 2, Table S2 and S3).

The other analyses, including differences in expression between male and female patients, between those who underwent total versus subtotal gastrectomy, EBV status, and whether or not patients received neoadjuvant therapy, showed no significant differences in the expression of the *MMPs* evaluated in this study (Figure 2).

**Figure 3:**
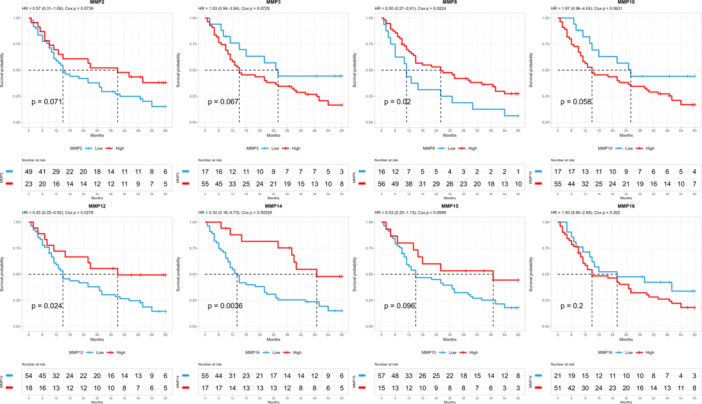
Influence of *MMP* expression alterations on patient survival. Comparison of the expression levels of *MMP2, MMP3, MMP8, MMP10, MMP12, MMP14, MMP15*, and *MMP16* genes at different probabilities of survival among analyzed patients. With statistical significance of padj < 0.05.

Among *MMPs* with significantly different expression compared to peritumoral tissue, low expression of *MMP8*, *MMP12*, and *MMP14* was associated with reduced patient survival (Figure 3 and Table S4). Furthermore, survival data from the TCGA‐STAD cohort were analyzed to validate the finding (Figure S1 and Table S5).

### 3.3 Correlation Between Genes in Gastric Cancer and Enrichment Pathways

**Figure 4:**
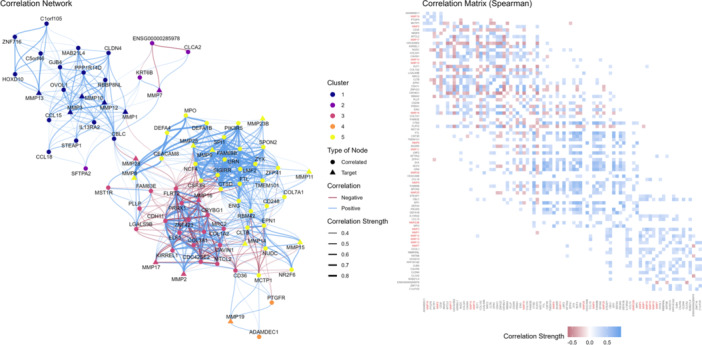
Correlations of differentially expressed *MMPs* with other genes in the genome. On the right, the correlation matrix highlights correlated genes, with negatively correlated genes shown in red and positively correlated genes in blue. On the left, the gene correlation network displays strongly correlated genes represented as nodes connected by edges in blue for positive correlation, and red for negative correlation, with edge thickness proportional to the strength of the association. Each co‐expressed gene group is indicated by distinct node colors. Nodes are classified as common genes (circles) or key genes (triangles).

The differentially expressed *MMP* between cancerous and non‐cancerous tissues were correlated with all other genes in the genome. Through this extensive analysis, significant expression correlations were detected between the *MMP2* gene and the genes *COL1A2* (*r* = 0.643), *CDH11* (*r* = 0.559), *CAVIN1* (*r* = 0.586), and *KIRREL1* (*r* = 0.584); *MMP3* gene and the genes *MMP12* (*r* = 0.674), *PPP1R14D* (*r* = 0.5131), *MAB21L4* (*r* = 0.533); *MMP8* gene and the genes *CEACAM8* (*r* = 0.720), *DEFA1B* (*r* = 0.713), *DEFA4* (*r* = 0.679), *MPO* (*r* = 0.660); *MMP10* gene and the genes *RBBP8NL* (*r* = 0.520), *IL13RA2* (*r* = 0.510), *CCL15* (*r* = 0.502); *MMP12* gene and the genes *MMP3* (*r* = 0.674), *CBLC* (*r* = 0.545), *PPP1R14D* (*r* = 0.575), *STEAP1* (*r* = 0.503), *MMP14* gene and the *RVM42* gene (*r* = 0.501), *MMP16* gene and the genes *ZNF423* (*r* = 0.788), *PRRX1* (*r* = 0.778), *CDH11* (*r* = 0.771), *FLRT2* (*r* = 0.748), *COL1A2* (*r* = 0.595) (Figure 4).

The correlation network analysis identified five co‐expressed groups, represented by distinct colors, that were significantly associated with differentially expressed *MMPs*. These associations reflect specific correlation patterns, with strongly correlated genes tending to cluster in close proximity within the network, forming groups that may represent shared biological pathways or processes (Figure 4).

**Figure 5:**
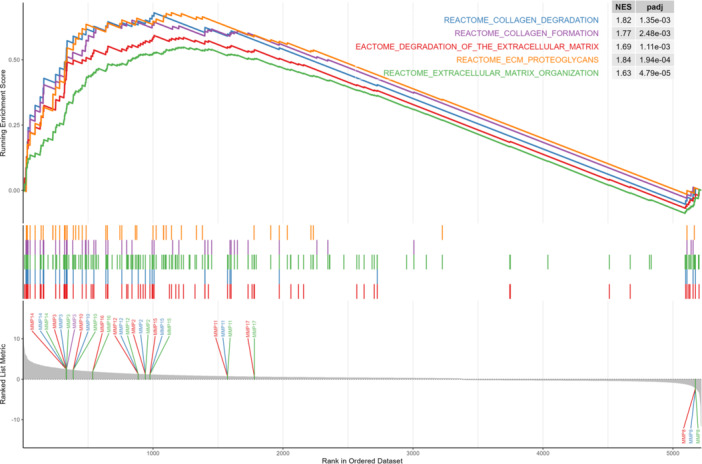
Enrichment analysis of pathways related to the *MMP* gene set. The plot displays the enrichment curves for the five major pathways significantly associated with *MMP* genes and their correlated partners, according to the REACTOME database: Collagen Degradation, Collagen Formation, Degradation of the Extracellular Matrix, ECM Proteoglycans, and Extracellular Matrix Organization. The colored ticks in the central panel represent the positions of pathway genes within the ranked list, illustrating their contribution to the observed enrichment pattern. Genes were ranked and used to calculate the normalized enrichment score (NES).

The enrichment pathways were generated from genes selected based on their correlation with *MMPs*, which showed significantly altered expression. Functionally enriched pathways of the 10 *MMP* genes with altered expression were determined using the REACTOME database. Additionally, annotated keywords were identified. A total of 93 biological pathways were identified as being strongly associated with *MMP* genes and their correlated genes (Supporting Material: Table 6). Five of these 93 pathways were selected for further analysis, as they show a strong biological relationship with the functions performed by the *MMPs* exhibiting altered expression in our samples. The selected pathways were: extracellular matrix organization, proteoglycan matrix, collagen degradation, collagen formation, and extracellular matrix degradation (Figure 5).

## 4 Discussion

MMPs are enzymes that cleave virtually all ECM proteins, as well as various non‐matrix proteins, thus directly participating in remodeling the stromal region of the stomach [31, 32, 33, 34, 35]. Therefore, modulation of *MMP* expression is not only associated with cancer invasion and metastasis but also with all stages of the carcinogenic process, from the earliest phases of epithelial transformation [36, 37, 38, 39].

Among the *MMPs* analyzed, eight genes (*MMP2*, *MMP3*, *MMP8, MMP10*, *MMP12*, *MMP14*, *MMP15*, *MMP16*) were found to be differentially expressed in tumor tissue compared to peritumoral tissue. Except *MMP8*, which showed reduced expression in tumor tissue and has previously been described as a potential tumor suppressor [40], all other genes showed increased expression in gastric cancer and are considered potential tumor biomarkers, in addition to contributing directly and/or indirectly to tumor progression [41, 42, 43, 44, 45, 46, 47, 48, 49].

These findings suggest that the tumor microenvironment in gastric cancer is modulated through multiple mechanisms, involving distinct molecular pathways and biological processes. For instance, MMP2 and MMP14 play a central role in ECM remodeling, where MMP2, directly activated by MMP14, degrades type IV collagen, which, together with type I collagen, is highly abundant in gastric tumor microenvironments, particularly in tumors with extensive fibroblast recruitment [50, 51]. In addition to MMP14, the MMP15, and MMP16 also contribute to pro‐MMP2 activation, thereby promoting ECM remodeling, particularly of the basement membrane, and facilitating the early stages of tumor invasion [52].

A previous in vitro study demonstrated that gastric adenocarcinoma (AGS) cells, when treated with ouabain, a steroid hormone, exhibit inhibition of *MMP2* production, resulting in a significant reduction in invasion and metastasis processes [53]. These findings indicate that *MMP2*, which is also altered in the samples of the present study, plays an important role in cancer progression and may represent a potential therapeutic target.

An analysis was performed to assess the stability of constitutive (reference) genes across the evaluated samples (Tables S7 and S8). The results demonstrated that these genes exhibited low expression variability among samples, indicating appropriate data normalization and technical consistency of the analyses. These findings support the reliability of the results obtained from the analyzed cohort.

Based on these observations, we hypothesized that the increased expression of *MMPs* observed in the early stages of the disease may be associated with molecular characteristics specific to the studied population. For example, certain communities from Northern Brazil present a higher frequency of germline molecular alterations, which may contribute to carcinogenesis and influence disease prognosis when compared with populations lacking such genetic features [54, 55].

In our dataset, higher expression levels of *MMP2, MMP3, MMP10, MMP12, MMP14*, and *MMP15* were observed in stages TNM II and III compared with stage TNM IV. This expression pattern may be associated with tumor heterogeneity and region‐specific molecular features, suggesting that patients from this population may exhibit increased susceptibility to invasive and metastatic processes at earlier stages of the disease.

Additionally, we performed a survival analysis using the TCGA‐STAD cohort to validate our findings. Differences in prognostic behavior were observed between the two cohorts. Notably, *MMP8, MMP12*, and *MMP14* genes were associated with poorer overall survival in our cohort. The Kaplan–Meier curves showed crossing patterns in the TCGA‐STAD cohort, indicating distinct prognostic effects between populations.

It is important to note that the TCGA‐STAD cohort is composed predominantly of patients from North America and Europe, as reported by the TCGA consortium (TCGA, 2014), whereas our cohort consists of patients from South America, specifically from Northern Brazil. This distinction reinforces the hypothesis that patients from Northern Brazil present unique molecular characteristics that may directly influence gene expression profiles, tumor behavior, and clinical outcomes.

These findings suggest that alterations in *MMP* expression may influence tumor progression and survival. These findings are consistent with evidence reported in previous clinical studies that investigated a broad‐spectrum, low–molecular‐weight MMP inhibitor, which showed beneficial effects during the early phases of clinical trials by increasing patient survival [56, 57]. Collectively, these findings highlight the importance of the *MMP* family in the biology of gastric cancer.

Other *MMPs* involved in gastric cancer progression include *MMP3* and *MMP10*. These genes promote the degradation of similar substrates, such as type III, IV, and V collagens, proteoglycans, and laminins. Moreover, along with *MMP12*, they contribute both directly and indirectly to inflammatory processes within the tumor microenvironment, as their enzymes can be secreted by immune cells, such as macrophages, and can also be induced by inflammatory cytokines [58, 59, 60, 61].

Due to the high expression of *MMP3, MMP10*, and *MMP12* in the investigated samples, and their association with immune system cells, it is likely that M2 macrophage activation occurs in the tumor microenvironment, which may promote immunosuppression, suggesting a possible tumor adaptive immune regulation during the progression of GC [61, 62].

Paradoxically, when we analyzed *MMP8*, its expression was decreased in gastric tumor tissues, unlike the other *MMPs* with altered expression compared to non‐tumorous tissue. However, it has been reported that in some types of cancer, this gene, when expressed at high levels, has an antitumor action, in addition to being associated with the modulation of the tumor inflammatory response [63, 64, 65].

Additionally, we analyzed the correlated genes, along with the enrichment of biological pathways involving the *MMP* family. The differentially expressed *MMPs* were associated with genes responsible for adhesion, ECM organization, and immune system modulation. The genetic correlation network analysis demonstrated the presence of five distinct co‐expressed clusters, suggesting a functional compartmentalization of *MMPs* within specific biological pathways.

*MMP2* was shown to be positively correlated with the genes *COL1A2* and *CAVIN1*, which are associated with the production of collagen I and the regulation of genes important in carcinogenesis, such as *MMPs*, respectively [66, 67]. Furthermore, there was a correlation with the *CDH11* and *KIRREL1* genes, which are associated with cell adhesion and the maintenance of specialized junction integrity. Analysis in The Human Protein Atlas revealed that the KIRREL1 protein is among the top 15 proteins most strongly correlated with MMP2, which reinforces and corroborates our findings with the pattern described in the literature [68, 69]. When observing the correlation network, these genes are clustered, suggesting they are strongly associated and possibly related to collagen formation and, consequently, ECM organization, corroborating the biological pathway enrichment findings.

A strong positive correlation was also found between *MMP3* and *MMP12*, both responsible for ECM degradation, inflammation, tissue remodeling, and the carcinogenic process [70], reinforcing our pathway enrichment results. In addition to this association, *MMP3* and *MMP12* were also linked to the *PPP1R14D* gene, which is responsible for the indirect modulation of MMPs. In a lung cancer study, it was demonstrated that inhibition of the *PPP1R14D* gene may be associated with proliferative, migratory, and invasive cell activity, as it can inactivate, among various genes, members of the *MMP* family [71].

Regarding *MMP8*, the gene was found to correlate with genes primarily involved in the inflammatory response, such as *CEACAM8*—a cell‐cell communication molecule that also plays an immunoregulatory role, particularly in granulocyte activation [72]—as well as the genes *DEFA1B* and *DEFA4*, which have previously been described as prognostic biomarkers in lung cancer [73]. These associations highlight the potential influence of *MMP8* on the immune response.

Furthermore, *MMP16* was found to be correlated with the genes *ZNF423, PRRX1, CDH11, FLRT2*, and *COL1A2*, which are associated with the modulation of genes involved in invasion, as well as the promotion of migration and invasion. In the literature, the relationship between *MMP16* and the *ZNF423* gene remains unclear; however, ZNF423 indirectly modulates the extracellular matrix through the regulation of other MMPs [74]. We suggest that the association between MMP16 and PRRX1 may be related to ECM modulation, as PRRX1 acts as a master transcription factor for myofibroblasts [75]. Given that myofibroblasts are key players in the release of enzymes that modulate the ECM, this finding supports the observed association with the *COL1A2* gene, which is responsible for type I collagen production, as well as the relationship with the *FLRT2* gene, known to interact with fibroblast growth factors [76].

## 5 Conclusion

These findings provide further insights into disorders involving the *MMP* gene family, their interactions with other genes within the transcriptome, and the relationship of these alterations with clinicopathological features of patients from northern Brazil. We suggest that this set of genes plays a role in biological processes associated with proteolysis, particularly in the catabolism of collagens and proteoglycans, as well as in the disassembly of the extracellular matrix, processes that are modulated by MMPs proteins in gastric cancer. Although our findings provide important insights, the proposed hypotheses require investigation through functional studies and further validation.

## Author Contributions

**Aline Costa Bastos:** conceptualization, visualization, investigation, formal analysis, writing – original draft, writing – review and editing, validation. **André Salim Khayat:** writing – review and editing, writing – original draft, supervision, visualization. **Emanuele Raimunda Louzada Moraes:** writing – review and editing, writing – original draft, investigation, methodology. **Ágatha Tereza Miranda Tavares:** writing – review and editing, writing – original draft, investigation, methodology. **Ronald Matheus da Silva Mourão:** writing – review and editing, writing – original draft, data curation, formal analysis, methodology, software. **Fabiano Cordeiro Moreira:** writing – review and editing, writing – original draft, data curation, formal analysis, methodology, software, supervision. **Samir Mansour Moraes Casseb:** writing – review and editing, writing – original draft, data curation, formal analysis, methodology, investigation, supervision. **Samia Demachki:** writing – review and editing, writing – original draft, investigation, validation. **Geraldo Ishak:** writing – review and editing; writing – original draft, investigation, validation. **Williams Fernandes Barra:** writing – review and editing, writing – original draft, investigation, visualization. **Rommel Mario Rodríguez Burbano:** writing – review and editing, writing – original draft, conceptualization, funding acquisition, project administration; resources, visualization. **Paulo Pimentel de Assumpção:** writing – review and editing, writing – original draft, conceptualization, funding acquisition, supervision, project administration, resources, visualization.

## Conflicts of Interest

The authors declare no conflicts of interest.

## Supporting information


**Figure 1** ‐ Overall survival analysis of patients from the The Cancer Genome Atlas Stomach Adenocarcinoma (TCGA‐STAD) cohort. Comparison of the expression levels of *MMP2, MMP3, MMP8, MMP10, MMP12, MMP14, MMP15*, and *MMP16* genes at different probabilities of survival among analyzed patients. With statistical significance of padj < 0.05.

**Supporting Material Table 1** ‐ Analysis of differential gene expression in the matrix metalloproteinase (MMP) gene family.

**Supporting Material Table 2** ‐ Distribution of gastric cancer samples according to TCGA subtypes.

**Supporting Material Table 3** ‐ Differential expression analysis of *MMP* family genes across TCGA molecular classifications.

**Supporting Material Table 4** ‐ Median overall survival according to MMP expression levels (low vs. high).

**Supporting Material Table 5** ‐ Overall survival analysis of patients from the The Cancer Genome Atlas Stomach Adenocarcinoma (TCGA‐STAD) cohort, in *MMP* samples with significantly altered global expression.

**Supporting Material Table 6** ‐ Enrichment analysis of pathways related to the MMP gene set.

**Supporting Material Table 7** ‐ Analysis of the expression of housekeeping genes in samples from patients with gastric cancer.

**Supporting Material Table 8** ‐ Analysis of the expression of housekeeping genes in gastric cancer samples, with distribution in tumoral (CG) and peritumoral (PTT) regions.

## Data Availability

The data that supports the findings of this study are available in the Supporting Material of this article.
