# Supplementary material for: Analysis of the Matrix Metalloproteinases Family Profile in Gastric Cancer Suggests Key Matrix Metalloproteinases for Tumor Development and Their Clinical Impact
Source: Mol Carcinog. 2026 Feb 23;65(5):577–88. doi: 10.1002/mc.70097 (PMC13067799; doi:10.1002/mc.70097)
Supplement: Supplementary file 1 — Figure 1 ‐ Overall survival analysis of patients from the The Cancer Genome Atlas Stomach Adenocarcinoma (TCGA‐STAD) cohort. Comparison of the expression levels of MMP2, MMP3, MMP8, MMP10, MMP12, MMP14, MMP15, and MMP16 genes at different probabilities of survival among analyzed patients. With statistical significance of padj < 0.05. [file MC-65-577-s002.docx]

**Supplementary Material**


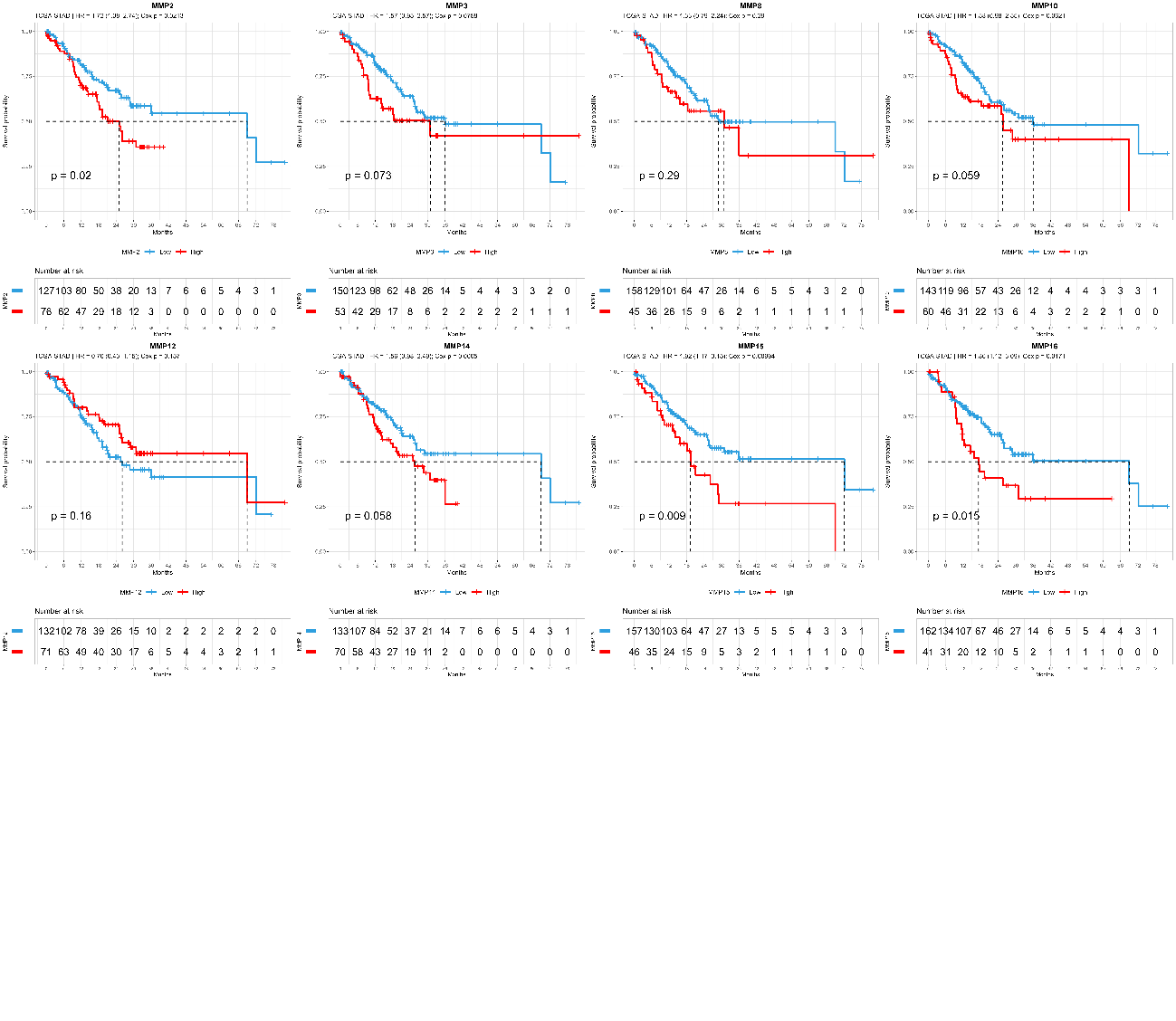


**Figure 1 - Overall survival analysis of patients from the The Cancer Genome Atlas Stomach Adenocarcinoma (TCGA-STAD) cohort.**Comparison of the expression levels of *MMP2, MMP3, MMP8, MMP10, MMP12, MMP14, MMP15,* and *MMP16* genes at different probabilities of survival among analyzed patients. With statistical significance of padj < 0.05.
