# Supplementary material for: Analysis of the Matrix Metalloproteinases Family Profile in Gastric Cancer Suggests Key Matrix Metalloproteinases for Tumor Development and Their Clinical Impact
Source: Mol Carcinog. 2026 Feb 23;65(5):577–88. doi: 10.1002/mc.70097 (PMC13067799; doi:10.1002/mc.70097)
Supplement: Supplementary file 2 — Supporting Material Table 1 ‐ Analysis of differential gene expression in the matrix metalloproteinase (MMP) gene family. [file MC-65-577-s007.docx]

**Supplementary Material Table 1 - Analysis of differential gene expression in the matrix metalloproteinase (MMP) gene family.**

| **Gene** | **baseMean** | **log2FoldChange** | **pvalue** | **padj** | **from_list** | **significant** |
| --- | --- | --- | --- | --- | --- | --- |
| MMP1 | 137.636785 | 0.7490847 | 0.02263106 | 0.06980024 | VERDADEIRO | FALSE |
| MMP2 | 184.840311 | 1.22979396 | 0.0011739 | 0.00648261 | VERDADEIRO | TRUE |
| MMP3 | 60.6767816 | 2.48450514 | 2.4474E-08 | 5.2162E-07 | VERDADEIRO | TRUE |
| MMP7 | 42.0029227 | -0.51200239 | 0.23988289 | 0.40838781 | VERDADEIRO | FALSE |
| MMP8 | 80.0691176 | -2.30381579 | 4.1038E-11 | 1.4566E-09 | VERDADEIRO | TRUE |
| MMP9 | 364.788822 | -0.04333151 | 0.85590798 | 0.93111461 | VERDADEIRO | FALSE |
| MMP10 | 10.5218896 | 2.30353472 | 4.6856E-08 | 9.3846E-07 | VERDADEIRO | TRUE |
| MMP11 | 14.6510497 | 0.73598833 | 0.00811015 | 0.03099181 | VERDADEIRO | FALSE |
| MMP12 | 47.7764367 | 1.28962347 | 0.0010852 | 0.00608049 | VERDADEIRO | TRUE |
| MMP13 | 2.39466841 | 0.49377589 | 0.48387105 | 0.66426621 | VERDADEIRO | FALSE |
| MMP14 | 186.758411 | 2.49205574 | 4.3632E-30 | 2.4004E-27 | VERDADEIRO | TRUE |
| MMP15 | 79.9923224 | 1.18444168 | 6.9247E-05 | 0.00059906 | VERDADEIRO | TRUE |
| MMP16 | 82.1056068 | 1.88985704 | 1.3221E-06 | 1.9269E-05 | VERDADEIRO | TRUE |
| MMP17 | 20.2942149 | 0.63709734 | 0.00158838 | 0.00822937 | VERDADEIRO | FALSE |
| MMP19 | 36.5642443 | 0.58703087 | 0.02991529 | 0.08689305 | VERDADEIRO | FALSE |
| MMP20 | 0.8373227 | 0.40939463 | 0.89865447 |  | VERDADEIRO | FALSE |
| MMP21 | 3.76369961 | -0.11208872 | 0.74427999 | 0.86488774 | VERDADEIRO | FALSE |
| MMP24 | 11.999961 | 0.67044156 | 0.07100502 | 0.16842407 | VERDADEIRO | FALSE |
| MMP25 | 303.828862 | 0.04208174 | 0.83159907 | 0.91927047 | VERDADEIRO | FALSE |
| MMP26 | 0.10054333 | 0.24421005 | 0.94495112 |  | VERDADEIRO | FALSE |
| MMP27 | 0.15306565 | -0.11729827 | 0.9168246 |  | VERDADEIRO | FALSE |
| MMP28 | 9.7427504 | 0.2125194 | 0.36353318 | 0.54733615 | VERDADEIRO | FALSE |
