# Supplementary material for: Analysis of the Matrix Metalloproteinases Family Profile in Gastric Cancer Suggests Key Matrix Metalloproteinases for Tumor Development and Their Clinical Impact
Source: Mol Carcinog. 2026 Feb 23;65(5):577–88. doi: 10.1002/mc.70097 (PMC13067799; doi:10.1002/mc.70097)
Supplement: Supplementary file 3 — Supporting Material Table 2 ‐ Distribution of gastric cancer samples according to TCGA subtypes. [file MC-65-577-s009.docx]

**Supplementary Material Table 2 - Distribution of gastric cancer samples according to TCGA subtypes.**

| **TCGA_classification** | **n** | **pct** |
| --- | --- | --- |
| CIN | 27 | 57.4 |
| MSI | 8 | 17 |
| EBV | 6 | 12.8 |
| GS | 6 | 12.8 |
