# Supplementary material for: Analysis of the Matrix Metalloproteinases Family Profile in Gastric Cancer Suggests Key Matrix Metalloproteinases for Tumor Development and Their Clinical Impact
Source: Mol Carcinog. 2026 Feb 23;65(5):577–88. doi: 10.1002/mc.70097 (PMC13067799; doi:10.1002/mc.70097)
Supplement: Supplementary file 4 — Supporting Material Table 3 ‐ Differential expression analysis of MMP family genes across TCGA molecular classifications. [file MC-65-577-s008.docx]

**Supplementary Material Table 3 - Differential expression analysis of MMP family genes across TCGA molecular classifications.**

| **BaseMean** | **log2FoldChange** | **pvalue** | **padj** | **Gene** | **contrast_info** |
| --- | --- | --- | --- | --- | --- |
| 7,946,208,577 | -1,148,605,008 | 0,03764210436 | 0,1951033963 | MMP2 | tcga_classification: MSI vs CIN |
| 2,523,098,789 | -1,098,742,569 | 0,05686792295 | 0,2490674831 | MMP16 | tcga_classification: MSI vs CIN |
| 2,053,180,386 | 1,588,958,533 | 0,07202589944 | 0,2866116751 | MMP3 | tcga_classification: MSI vs CIN |
| 3,023,784,934 | 1,292,676,998 | 0,08951351415 | 0,3223306985 | MMP12 | tcga_classification: MSI vs CIN |
| 5,386,137,952 | 0,7956357513 | 0,2846933118 | 0,5912670447 | MMP10 | tcga_classification: MSI vs CIN |
| 2,517,109,785 | -0,2385014621 | 0,4887307405 | 0,7539977408 | MMP15 | tcga_classification: MSI vs CIN |
| 3,165,295,066 | -0,2430580922 | 0,8300844251 | 0,9362697487 | MMP8 | tcga_classification: MSI vs CIN |
| 7,101,134,452 | -0,008872390309 | 0,9788850388 | 0,9921332857 | MMP14 | tcga_classification: MSI vs CIN |
| 3,023,784,934 | 1,945,973,076 | 0,05746421522 | 0,3175016946 | MMP12 | tcga_classification: MSI vs GS |
| 7,946,208,577 | -1,066,433,166 | 0,1498233619 | 0,5036698272 | MMP2 | tcga_classification: MSI vs GS |
| 2,523,098,789 | -0,7364348299 | 0,3346167302 | 0,6922940652 | MMP16 | tcga_classification: MSI vs GS |
| 5,386,137,952 | -0,9442524406 | 0,3399360865 | 0,695742814 | MMP10 | tcga_classification: MSI vs GS |
| 2,517,109,785 | 0,3291947742 | 0,4776092968 | 0,7870955614 | MMP15 | tcga_classification: MSI vs GS |
| 7,101,134,452 | -0,0966079756 | 0,8298250782 | 0,9511729636 | MMP14 | tcga_classification: MSI vs GS |
| 2,053,180,386 | 0,2551373081 | 0,8294791461 | 0,9511729636 | MMP3 | tcga_classification: MSI vs GS |
| 3,165,295,066 | 0,1074925689 | 0,9440289409 | 0,9865201634 | MMP8 | tcga_classification: MSI vs GS |
| 5,386,137,952 | -3,244,173,778 | 0,0009531390277 | 0,01987193728 | MMP10 | tcga_classification: MSI vs EBV |
| 2,523,098,789 | -1,213,403,193 | 0,1111028856 | 0,3414118086 | MMP16 | tcga_classification: MSI vs EBV |
| 7,946,208,577 | -0,7114868644 | 0,3367022446 | 0,612190227 | MMP2 | tcga_classification: MSI vs EBV |
| 7,101,134,452 | -0,4287222869 | 0,3397756466 | 0,6144869847 | MMP14 | tcga_classification: MSI vs EBV |
| 3,023,784,934 | -0,5675568017 | 0,5781244601 | 0,7942694813 | MMP12 | tcga_classification: MSI vs EBV |
| 2,053,180,386 | -0,5296593936 | 0,6546857816 | 0,8371434514 | MMP3 | tcga_classification: MSI vs EBV |
| 2,517,109,785 | -0,1690939138 | 0,713950165 | 0,8690843178 | MMP15 | tcga_classification: MSI vs EBV |
