# Supplementary material for: Analysis of the Matrix Metalloproteinases Family Profile in Gastric Cancer Suggests Key Matrix Metalloproteinases for Tumor Development and Their Clinical Impact
Source: Mol Carcinog. 2026 Feb 23;65(5):577–88. doi: 10.1002/mc.70097 (PMC13067799; doi:10.1002/mc.70097)
Supplement: Supplementary file 5 — Supporting Material Table 4 ‐ Median overall survival according to MMP expression levels (low vs. high). [file MC-65-577-s001.docx]

**Supplementary Material Table 4 - Median overall survival according to MMP expression levels (low vs. high)**

| **Gene** | **Group** | **n** | **Events** | **Median_survival** | **Median_followup** |
| --- | --- | --- | --- | --- | --- |
| MMP2 | Low | 49 | 40 | 15 | 15 |
| MMP2 | High | 23 | 14 | 39 | 39 |
| MMP3 | Low | 17 | 9 | 32 | 31 |
| MMP3 | High | 55 | 45 | 15 | 15 |
| MMP8 | Low | 16 | 15 | 11 | 11 |
| MMP8 | High | 56 | 39 | 26 | 26 |
| MMP10 | Low | 17 | 9 | 32 | 31 |
| MMP10 | High | 55 | 45 | 15 | 15 |
| MMP12 | Low | 54 | 45 | 15 | 15 |
| MMP12 | High | 18 | 9 | 39 | 38 |
| MMP14 | Low | 55 | 46 | 14 | 14 |
| MMP14 | High | 17 | 8 | 49 | 40 |
| MMP15 | Low | 57 | 46 | 15 | 15 |
| MMP15 | High | 15 | 8 | 49 | 37 |
| MMP16 | Low | 21 | 13 | 26 | 26 |
| MMP16 | High | 51 | 41 | 15 | 15 |
|  |  |  |  |  |  |
