# Supplementary material for: Analysis of the Matrix Metalloproteinases Family Profile in Gastric Cancer Suggests Key Matrix Metalloproteinases for Tumor Development and Their Clinical Impact
Source: Mol Carcinog. 2026 Feb 23;65(5):577–88. doi: 10.1002/mc.70097 (PMC13067799; doi:10.1002/mc.70097)
Supplement: Supplementary file 6 — Supporting Material Table 5 ‐ Overall survival analysis of patients from the The Cancer Genome Atlas Stomach Adenocarcinoma (TCGA‐STAD) cohort, in MMP samples with significantly altered global expression. [file MC-65-577-s004.docx]

**Supplementary Material Table 5 - Overall survival analysis of patients from the The Cancer Genome Atlas Stomach Adenocarcinoma (TCGA-STAD) cohort, in *MMP* samples with significantly altered global expression**

| **Cohort** | **gene** | **hr_high_vs_low** | **ci_low** | **ci_high** | **cox_p** | **direction** | **n_low** | **n_high** | **events**  **low** | **Events**  **high** |
| --- | --- | --- | --- | --- | --- | --- | --- | --- | --- | --- |
| TCGA-STAD | MMP2 | 1.72287225 | 1.084299 | 2.73752 | 0.021303 | High worse (HR>1) | 127 | 76 | 38 | 36 |
| TCGA-STAD | MMP3 | 1.565330795 | 0.954599 | 2.566796 | 0.075756 | High worse (HR>1) | 150 | 53 | 51 | 23 |
| TCGA-STAD | MMP8 | 1.325559487 | 0.786073 | 2.235298 | 0.290449 | High worse (HR>1) | 158 | 45 | 55 | 19 |
| TCGA-STAD | MMP10 | 1.578594977 | 0.97708 | 2.550418 | 0.062144 | High worse (HR>1) | 143 | 60 | 48 | 26 |
| TCGA-STAD | MMP12 | 0.704112583 | 0.432945 | 1.14512 | 0.157401 | High better (HR<1) | 132 | 71 | 49 | 25 |
| TCGA-STAD | MMP14 | 1.561696214 | 0.980506 | 2.487385 | 0.060504 | High worse (HR>1) | 133 | 70 | 42 | 32 |
| TCGA-STAD | MMP15 | 1.917325731 | 1.168822 | 3.145165 | 0.009944 | High worse (HR>1) | 157 | 46 | 51 | 23 |
| TCGA-STAD | MMP16 | 1.858012189 | 1.116818 | 3.091113 | 0.01706 | High worse (HR>1) | 162 | 41 | 53 | 21 |
