# Supplementary material for: Analysis of the Matrix Metalloproteinases Family Profile in Gastric Cancer Suggests Key Matrix Metalloproteinases for Tumor Development and Their Clinical Impact
Source: Mol Carcinog. 2026 Feb 23;65(5):577–88. doi: 10.1002/mc.70097 (PMC13067799; doi:10.1002/mc.70097)
Supplement: Supplementary file 7 — Supporting Material Table 6 ‐ Enrichment analysis of pathways related to the MMP gene set. [file MC-65-577-s003.docx]

**Supplementary Material Table 6 -  Enrichment analysis of pathways related to the MMP gene set**

| **ID** | **Description** | **setSize** | **enrichmentScore** | **NES** | **pvalue** | **p.adjust** | **qvalue** | **rank** | **leading_edge** | **core_enrichment** |
| --- | --- | --- | --- | --- | --- | --- | --- | --- | --- | --- |
| REACTOME_  ANTIMICROBIAL_PEPTIDES | REACTOME_ANTIMICROBIAL_PEPTIDES | 24 | -0.84267 | -2.8759 | 1E-10 | 7.35E-08 | 6.06E-08 | 198 | tags=79%, list=4%, signal=77% | DEFA1B/CAMP/PGLYRP1/ELANE/BPIFB1/LCN2/BPI/CTSG/DEFB1/RNASE3/DEFA4/PRTN3/CHGA/LTF/DEFB4A/DEFA3/DEFA5/DEFA1/DEFA6 |
| REACTOME_  MEIOTIC_RECOMBINATION | REACTOME_MEIOTIC_RECOMBINATION | 24 | -0.80717 | -2.75474 | 4.13E-09 | 1.39E-06 | 1.15E-06 | 518 | tags=83%, list=10%, signal=75% | H4C6/H2BC5/BLM/H4C4/H2AC14/H2BC9/H2BC17/H4C3/H4C13/H2BC6/H3C3/H3C11/H2BC3/H3C8/RAD51/H2BC13/H3C2/H3C15/H3C12/H3C7 |
| REACTOME_CONDENSATION_OF_PROPHASE_CHROMOSOMES | REACTOME_CONDENSATION_OF_PROPHASE_CHROMOSOMES | 28 | -0.76683 | -2.72624 | 5.68E-09 | 1.39E-06 | 1.15E-06 | 518 | tags=82%, list=10%, signal=74% | SMC2/H4C8/H4C6/H2BC5/CCNB1/H4C4/H2AC14/H2BC9/PLK1/H2BC17/H4C3/H4C13/H2BC6/H3C3/H3C11/H2BC3/H3C8/H2BC13/H3C2/H3C15/H3C12/H3C7/CDK1 |
| REACTOME_ASSEMBLY_OF_THE_ORC_COMPLEX_AT_THE_ORIGIN_OF_REPLICATION | REACTOME_ASSEMBLY_OF_THE_ORC_COMPLEX_AT_THE_ORIGIN_OF_REPLICATION | 28 | -0.75769 | -2.69378 | 1.35E-08 | 2.49E-06 | 2.05E-06 | 518 | tags=71%, list=10%, signal=65% | H4C6/H2BC5/ORC6/H4C4/H2AC14/H2BC9/H2BC17/H4C3/H4C13/H2BC6/H3C3/ORC1/H3C11/H2BC3/H3C8/H2BC13/H3C2/H3C15/H3C12/H3C7 |
| REACTOME_CELL_CYCLE_CHECKPOINTS | REACTOME_CELL_CYCLE_CHECKPOINTS | 89 | -0.51559 | -2.23038 | 3.08E-08 | 4.52E-06 | 3.73E-06 | 741 | tags=48%, list=14%, signal=42% | DNA2/H4C8/CENPL/CENPU/NDC80/CENPE/H4C6/RMI1/H2BC5/BLM/CCNB1/ORC6/H4C4/H2BC9/PLK1/H2BC17/CCNE2/KNL1/BUB1B/H4C3/H4C13/H2BC6/ORC1/ERCC6L/H2BC3/BUB1/CCNA2/GTSE1/CDCA8/CDC25A/KIF2C/CENPF/PKMYT1/H2BC13/CENPA/AURKB/CENPI/CLSPN/CDK1/CCNB2/MCM10/SKA1/BIRC5 |
| REACTOME_DEPOSITION_OF_NEW_CENPA_CONTAINING_NUCLEOSOMES_AT_THE_CENTROMERE | REACTOME_DEPOSITION_OF_NEW_CENPA_CONTAINING_NUCLEOSOMES_AT_THE_CENTROMERE | 22 | -0.78753 | -2.67821 | 7.08E-08 | 7.94E-06 | 6.55E-06 | 738 | tags=86%, list=14%, signal=74% | H4C8/CENPL/CENPU/H4C6/H2BC5/H4C4/H2AC14/H2BC9/H2BC17/KNL1/H4C3/H4C13/H2BC6/H2BC3/HJURP/H2BC13/CENPA/CENPI/OIP5 |
| REACTOME_CHROMATIN_MODIFICATIONS_DURING_THE_MATERNAL_TO_ZYGOTIC_TRANSITION_MZT | REACTOME_CHROMATIN_MODIFICATIONS_DURING_THE_MATERNAL_TO_ZYGOTIC_TRANSITION_MZT | 25 | -0.7432 | -2.57283 | 7.57E-08 | 7.94E-06 | 6.55E-06 | 518 | tags=72%, list=10%, signal=65% | H4C6/H2BC5/H4C4/H2AC14/H2BC9/H2BC17/H4C3/H4C13/H2BC6/H3C3/H3C11/H2BC3/H3C8/H2BC13/H3C2/H3C15/H3C12/H3C7 |
| REACTOME_DEVELOPMENTAL_LINEAGE_OF_PANCREATIC_DUCTAL_CELLS | REACTOME_DEVELOPMENTAL_LINEAGE_OF_PANCREATIC_DUCTAL_CELLS | 28 | 0.791242 | 2.034212 | 1.08E-07 | 9.89E-06 | 8.16E-06 | 797 | tags=79%, list=15%, signal=67% | COL11A1/COL1A1/COL1A2/COL3A1/COL2A1/FN1/NKX6-1/LAMA4/COL5A2/YAP1/SOX9/LAMB1/LAMA5/KRT19/LAMA1/COL5A1/CFTR/LAMC3/FOXA2/AQP1/SLC4A4/LAMC2 |
| REACTOME_DNA_REPLICATION | REACTOME_DNA_REPLICATION | 54 | -0.59596 | -2.30725 | 1.7E-07 | 1.39E-05 | 1.14E-05 | 804 | tags=56%, list=15%, signal=47% | RBX1/DNA2/H4C8/GMNN/H4C6/GINS3/H2BC5/ORC6/H4C4/H2AC14/POLE2/H2BC9/H2BC17/CCNE2/H4C3/H4C13/H2BC6/H3C3/ORC1/H3C11/H2BC3/H3C8/CCNA2/GINS2/H2BC13/H3C2/H3C15/H3C12/H3C7/MCM10 |
| REACTOME_TRANSCRIPTIONAL_REGULATION_OF_GRANULOPOIESIS | REACTOME_TRANSCRIPTIONAL_REGULATION_OF_GRANULOPOIESIS | 31 | -0.68256 | -2.42164 | 2.31E-07 | 1.7E-05 | 1.4E-05 | 518 | tags=68%, list=10%, signal=61% | H4C6/GFI1/H2BC5/H4C4/H2AC14/H2BC9/H2BC17/H4C3/H4C13/H2BC6/H3C3/MYB/H3C11/H2BC3/H3C8/H2BC13/H3C2/H3C15/H3C12/CEBPE/H3C7 |
| REACTOME_DNA_METHYLATION | REACTOME_DNA_METHYLATION | 23 | -0.77606 | -2.67615 | 5.35E-07 | 3.57E-05 | 2.95E-05 | 518 | tags=78%, list=10%, signal=71% | H4C6/H2BC5/H4C4/H2AC14/H2BC9/H2BC17/H4C3/H4C13/H2BC6/H3C3/H3C11/H2BC3/H3C8/H2BC13/H3C2/H3C15/H3C12/H3C7 |
| REACTOME_EXTRACELLULAR_MATRIX_ORGANIZATION | REACTOME_EXTRACELLULAR_MATRIX_ORGANIZATION | 166 | 0.548563 | 1.639402 | 5.94E-07 | 3.64E-05 | 3E-05 | 1234 | tags=58%, list=24%, signal=46% | FBN3/COL11A1/BGN/COL1A1/COL1A2/COL16A1/COL3A1/COL2A1/COL8A1/JAM2/FN1/COL6A1/COL12A1/BMP7/LAMA4/FMOD/FBLN1/COL4A1/ELN/ITGA10/COL5A2/MFAP2/SCUBE1/COL4A2/LAMB1/BCAN/MMP14/MMP3/LAMA5/COL6A2/EMILIN3/ITGB4/MMP10/LUM/SNTG2/ITGA11/AGRN/LAMA1/NID2/SNTA1/COL9A3/DDR1/ADAMTS2/EMILIN1/COL5A1/ADAM12/P3H3/KLK7/MMP16/COL7A1/PCOLCE/LAMC3/MFAP4/COL6A3/SDC2/LTBP2/ACAN/COL8A2/LTBP4/BMP4/TGFB3/COL9A1/LAMC2/SH3PXD2A/HTRA1/EFEMP2/LTBP3/COL23A1/SDC3/LRP4/APP/MMP12/PDGFB/A2M/SPP1/MMP2/MMP15/COL10A1/DCN/COL18A1/ITGA7/ITGB8/PTPRS/TGFB2/DDR2/FBLN2/ASPN/ADAMTS9/HSPG2/SERPINH1/ICAM1/CDH1/ACTG2/TLL1/SPARC/ADAMTS4 |
| REACTOME_SIRT1_NEGATIVELY_REGULATES_RRNA_EXPRESSION | REACTOME_SIRT1_NEGATIVELY_REGULATES_RRNA_EXPRESSION | 23 | -0.76226 | -2.62856 | 1.44E-06 | 8.12E-05 | 6.7E-05 | 518 | tags=78%, list=10%, signal=71% | H4C6/H2BC5/H4C4/H2AC14/H2BC9/H2BC17/H4C3/H4C13/H2BC6/H3C3/H3C11/H2BC3/H3C8/H2BC13/H3C2/H3C15/H3C12/H3C7 |
| REACTOME_PRC2_METHYLATES_HISTONES_AND_DNA | REACTOME_PRC2_METHYLATES_HISTONES_AND_DNA | 27 | -0.71685 | -2.52678 | 1.59E-06 | 8.37E-05 | 6.9E-05 | 518 | tags=67%, list=10%, signal=60% | H4C6/H2BC5/H4C4/H2AC14/H2BC9/H2BC17/H4C3/H4C13/H2BC6/H3C3/H3C11/H2BC3/H3C8/H2BC13/H3C2/H3C15/H3C12/H3C7 |
| REACTOME_ACTIVATED_PKN1_STIMULATES_TRANSCRIPTION_OF_AR_ANDROGEN_RECEPTOR_REGULATED_GENES_KLK2_AND_KLK3 | REACTOME_ACTIVATED_PKN1_STIMULATES_TRANSCRIPTION_OF_AR_ANDROGEN_RECEPTOR_REGULATED_GENES_KLK2_AND_KLK3 | 25 | -0.69489 | -2.40558 | 1.79E-06 | 8.75E-05 | 7.22E-05 | 518 | tags=72%, list=10%, signal=65% | H4C6/H2BC5/H4C4/H2AC14/H2BC9/H2BC17/H4C3/H4C13/H2BC6/H3C3/H3C11/H2BC3/H3C8/H2BC13/H3C2/H3C15/H3C12/H3C7 |
| REACTOME_REGULATION_OF_ENDOGENOUS_RETROELEMENTS_BY_THE_HUMAN_SILENCING_HUB_HUSH_COMPLEX | REACTOME_REGULATION_OF_ENDOGENOUS_RETROELEMENTS_BY_THE_HUMAN_SILENCING_HUB_HUSH_COMPLEX | 29 | -0.68187 | -2.42164 | 3.64E-06 | 0.000149 | 0.000123 | 819 | tags=72%, list=16%, signal=61% | TASOR/H4C8/RBM7/H4C6/H2BC5/H4C4/H2AC14/H2BC9/H2BC17/H4C3/H4C13/H2BC6/H3C3/H3C11/H2BC3/H3C8/H2BC13/H3C2/H3C15/H3C12/H3C7 |
| REACTOME_DNA_REPLICATION_PRE_INITIATION | REACTOME_DNA_REPLICATION_PRE_INITIATION | 45 | -0.60669 | -2.29638 | 3.56E-06 | 0.000149 | 0.000123 | 518 | tags=53%, list=10%, signal=48% | H4C8/GMNN/H4C6/H2BC5/ORC6/H4C4/H2AC14/POLE2/H2BC9/H2BC17/H4C3/H4C13/H2BC6/H3C3/ORC1/H3C11/H2BC3/H3C8/H2BC13/H3C2/H3C15/H3C12/H3C7/MCM10 |
| REACTOME_G2_M_CHECKPOINTS | REACTOME_G2_M_CHECKPOINTS | 48 | -0.58657 | -2.2409 | 3.66E-06 | 0.000149 | 0.000123 | 518 | tags=48%, list=10%, signal=44% | H4C6/RMI1/H2BC5/BLM/CCNB1/ORC6/H4C4/H2BC9/H2BC17/H4C3/H4C13/H2BC6/ORC1/H2BC3/CCNA2/GTSE1/CDC25A/PKMYT1/H2BC13/CLSPN/CDK1/CCNB2/MCM10 |
| REACTOME_MET_ACTIVATES_PTK2_SIGNALING | REACTOME_MET_ACTIVATES_PTK2_SIGNALING | 22 | 0.784501 | 1.955124 | 4.44E-06 | 0.000166 | 0.000137 | 564 | tags=64%, list=11%, signal=57% | COL11A1/COL1A1/COL1A2/COL3A1/MET/COL2A1/FN1/LAMA4/COL5A2/LAMB1/LAMA5/LAMA1/COL5A1/LAMC3 |
| REACTOME_MET_PROMOTES_CELL_MOTILITY | REACTOME_MET_PROMOTES_CELL_MOTILITY | 26 | 0.76287 | 1.9518 | 4.51E-06 | 0.000166 | 0.000137 | 564 | tags=54%, list=11%, signal=48% | COL11A1/COL1A1/COL1A2/COL3A1/MET/COL2A1/FN1/LAMA4/COL5A2/LAMB1/LAMA5/LAMA1/COL5A1/LAMC3 |
| REACTOME_ECM_PROTEOGLYCANS | REACTOME_ECM_PROTEOGLYCANS | 45 | 0.680555 | 1.856625 | 7.6E-06 | 0.000266 | 0.000219 | 1142 | tags=73%, list=22%, signal=58% | BGN/COL1A1/COL1A2/COL3A1/COL2A1/FN1/COL6A1/LAMA4/FMOD/COL4A1/COL5A2/COL4A2/LAMB1/BCAN/LAMA5/COL6A2/LUM/AGRN/LAMA1/COL9A3/COL5A1/COL6A3/ACAN/TGFB3/COL9A1/LRP4/APP/DCN/ITGA7/PTPRS/TGFB2/ASPN/HSPG2 |
| REACTOME_COLLAGEN_CHAIN_TRIMERIZATION | REACTOME_COLLAGEN_CHAIN_TRIMERIZATION | 28 | 0.726079 | 1.866683 | 1.7E-05 | 0.000568 | 0.000469 | 1008 | tags=79%, list=19%, signal=64% | COL11A1/COL1A1/COL1A2/COL16A1/COL3A1/COL2A1/COL8A1/COL6A1/COL12A1/COL4A1/COL5A2/COL4A2/COL6A2/COL9A3/COL5A1/COL7A1/COL6A3/COL8A2/COL9A1/COL23A1/COL10A1/COL18A1 |
| REACTOME_GENE_SILENCING_BY_RNA | REACTOME_GENE_SILENCING_BY_RNA | 41 | -0.57411 | -2.13071 | 2.03E-05 | 0.000649 | 0.000535 | 518 | tags=51%, list=10%, signal=47% | H4C6/H2BC5/BCDIN3D/H4C4/H2AC14/H2BC9/PIWIL4/H2BC17/H4C3/H4C13/H2BC6/H3C3/H3C11/H2BC3/ANG/H3C8/H2BC13/H3C2/H3C15/H3C12/H3C7 |
| REACTOME_DISEASES_OF_GLYCOSYLATION | REACTOME_DISEASES_OF_GLYCOSYLATION | 65 | 0.621647 | 1.76748 | 2.17E-05 | 0.000666 | 0.000549 | 1159 | tags=55%, list=22%, signal=44% | BGN/THSD4/ADAMTSL1/NOTCH3/PRELP/FMOD/MUC3A/BCAN/MUC17/THBS2/GPC1/MUC4/LUM/AGRN/SEMA5A/ADAMTS2/MUC5AC/MUC6/GPC6/ADAMTS7/GPC4/SDC2/ACAN/ADAMTSL5/ADAMTSL3/CSPG4/ADAMTSL2/SDC3/SEMA5B/ADAMTS12/DCN/SBSPON/ADAMTS10/ADAMTS9/HSPG2/B4GAT1 |
| REACTOME_HCMV_LATE_EVENTS | REACTOME_HCMV_LATE_EVENTS | 39 | -0.59674 | -2.1525 | 2.87E-05 | 0.000811 | 0.000669 | 518 | tags=54%, list=10%, signal=49% | H4C6/H2AC12/H2BC5/H2AC16/H4C4/H2AC14/H2BC9/H2BC17/H2AC13/H4C3/H4C13/H2BC6/H3C3/H3C11/H2BC3/H3C8/H2BC13/H3C2/H3C15/H3C12/H3C7 |
| REACTOME_COLLAGEN_DEGRADATION | REACTOME_COLLAGEN_DEGRADATION | 39 | 0.679912 | 1.82038 | 2.77E-05 | 0.000811 | 0.000669 | 1008 | tags=72%, list=19%, signal=58% | COL11A1/COL1A1/COL1A2/COL16A1/COL3A1/COL2A1/COL8A1/COL6A1/COL12A1/COL4A1/COL5A2/COL4A2/MMP14/MMP3/COL6A2/MMP10/COL9A3/COL5A1/COL7A1/COL6A3/COL8A2/COL9A1/COL23A1/MMP12/MMP2/MMP15/COL10A1/COL18A1 |
| REACTOME_MEIOSIS | REACTOME_MEIOSIS | 32 | -0.61049 | -2.17884 | 4.17E-05 | 0.001021 | 0.000843 | 518 | tags=62%, list=10%, signal=57% | H4C6/H2BC5/BLM/H4C4/H2AC14/H2BC9/H2BC17/H4C3/H4C13/H2BC6/H3C3/H3C11/H2BC3/H3C8/RAD51/H2BC13/H3C2/H3C15/H3C12/H3C7 |
| REACTOME_SCAVENGING_BY_CLASS_A_RECEPTORS | REACTOME_SCAVENGING_BY_CLASS_A_RECEPTORS | 13 | 0.825956 | 1.883571 | 4.11E-05 | 0.001021 | 0.000843 | 319 | tags=62%, list=6%, signal=58% | COL1A1/SCARA5/COL1A2/APOE/COL3A1/COLEC12/COL4A1/COL4A2 |
| REACTOME_COLLAGEN_FORMATION | REACTOME_COLLAGEN_FORMATION | 46 | 0.651793 | 1.786608 | 4.11E-05 | 0.001021 | 0.000843 | 838 | tags=57%, list=16%, signal=48% | COL11A1/COL1A1/COL1A2/COL16A1/COL3A1/COL2A1/COL8A1/COL6A1/COL12A1/COL4A1/COL5A2/COL4A2/MMP3/COL6A2/ITGB4/COL9A3/ADAMTS2/COL5A1/P3H3/COL7A1/PCOLCE/COL6A3/COL8A2/COL9A1/LAMC2/COL23A1 |
| REACTOME_DEGRADATION_OF_THE_EXTRACELLULAR_MATRIX | REACTOME_DEGRADATION_OF_THE_EXTRACELLULAR_MATRIX | 78 | 0.593073 | 1.70595 | 4.1E-05 | 0.001021 | 0.000843 | 1008 | tags=55%, list=19%, signal=45% | FBN3/COL11A1/COL1A1/COL1A2/COL16A1/COL3A1/COL2A1/COL8A1/FN1/COL6A1/COL12A1/COL4A1/ELN/COL5A2/SCUBE1/COL4A2/LAMB1/BCAN/MMP14/MMP3/LAMA5/COL6A2/MMP10/COL9A3/COL5A1/KLK7/MMP16/COL7A1/COL6A3/ACAN/COL8A2/COL9A1/LAMC2/HTRA1/COL23A1/MMP12/A2M/SPP1/MMP2/MMP15/COL10A1/DCN/COL18A1 |
| REACTOME_GASTRULATION | REACTOME_GASTRULATION | 34 | 0.69266 | 1.824603 | 4.69E-05 | 0.001089 | 0.000898 | 854 | tags=38%, list=16%, signal=32% | ZIC1/FOXC2/ZIC2/MSX1/FOXC1/OSR1/TCF7L1/YAP1/FOXA2/BMP4/TEAD2/FGFR1/FOXH1 |
| REACTOME_SIGNALING_BY_MET | REACTOME_SIGNALING_BY_MET | 39 | 0.672743 | 1.801188 | 4.74E-05 | 0.001089 | 0.000898 | 564 | tags=36%, list=11%, signal=32% | COL11A1/COL1A1/COL1A2/COL3A1/MET/COL2A1/FN1/LAMA4/COL5A2/LAMB1/LAMA5/LAMA1/COL5A1/LAMC3 |
| REACTOME_COLLAGEN_BIOSYNTHESIS_AND_MODIFYING_ENZYMES | REACTOME_COLLAGEN_BIOSYNTHESIS_AND_MODIFYING_ENZYMES | 38 | 0.672864 | 1.793652 | 5.67E-05 | 0.001263 | 0.001042 | 653 | tags=55%, list=13%, signal=49% | COL11A1/COL1A1/COL1A2/COL16A1/COL3A1/COL2A1/COL8A1/COL6A1/COL12A1/COL4A1/COL5A2/COL4A2/COL6A2/COL9A3/ADAMTS2/COL5A1/P3H3/COL7A1/PCOLCE/COL6A3/COL8A2 |
| REACTOME_MOLECULES_ASSOCIATED_WITH_ELASTIC_FIBRES | REACTOME_MOLECULES_ASSOCIATED_WITH_ELASTIC_FIBRES | 21 | 0.760878 | 1.877367 | 6.06E-05 | 0.001311 | 0.001081 | 1099 | tags=86%, list=21%, signal=68% | FBN3/FN1/BMP7/FBLN1/ELN/MFAP2/EMILIN3/EMILIN1/MFAP4/LTBP2/LTBP4/BMP4/TGFB3/EFEMP2/LTBP3/ITGB8/TGFB2/FBLN2 |
| REACTOME_ELASTIC_FIBRE_FORMATION | REACTOME_ELASTIC_FIBRE_FORMATION | 22 | 0.744929 | 1.856502 | 6.61E-05 | 0.001388 | 0.001145 | 1099 | tags=82%, list=21%, signal=65% | FBN3/FN1/BMP7/FBLN1/ELN/MFAP2/EMILIN3/EMILIN1/MFAP4/LTBP2/LTBP4/BMP4/TGFB3/EFEMP2/LTBP3/ITGB8/TGFB2/FBLN2 |
| REACTOME_ASSEMBLY_OF_COLLAGEN_FIBRILS_AND_OTHER_MULTIMERIC_STRUCTURES | REACTOME_ASSEMBLY_OF_COLLAGEN_FIBRILS_AND_OTHER_MULTIMERIC_STRUCTURES | 36 | 0.685596 | 1.813273 | 6.85E-05 | 0.001398 | 0.001153 | 797 | tags=61%, list=15%, signal=52% | COL11A1/COL1A1/COL1A2/COL3A1/COL2A1/COL8A1/COL6A1/COL12A1/COL4A1/COL5A2/COL4A2/MMP3/COL6A2/ITGB4/COL9A3/COL5A1/COL7A1/PCOLCE/COL6A3/COL8A2/COL9A1/LAMC2 |
| REACTOME_ERCC6_CSB_AND_EHMT2_G9A_POSITIVELY_REGULATE_RRNA_EXPRESSION | REACTOME_ERCC6_CSB_AND_EHMT2_G9A_POSITIVELY_REGULATE_RRNA_EXPRESSION | 29 | -0.62384 | -2.21556 | 7.5E-05 | 0.00149 | 0.001229 | 518 | tags=62%, list=10%, signal=56% | H4C6/H2BC5/H4C4/H2AC14/H2BC9/H2BC17/H4C3/H4C13/H2BC6/H3C3/H3C11/H2BC3/H3C8/H2BC13/H3C2/H3C15/H3C12/H3C7 |
| REACTOME_TRANSCRIPTIONAL_REGULATION_BY_SMALL_RNAS | REACTOME_TRANSCRIPTIONAL_REGULATION_BY_SMALL_RNAS | 33 | -0.59213 | -2.13163 | 9.85E-05 | 0.001905 | 0.001572 | 518 | tags=55%, list=10%, signal=49% | H4C6/H2BC5/H4C4/H2AC14/H2BC9/H2BC17/H4C3/H4C13/H2BC6/H3C3/H3C11/H2BC3/H3C8/H2BC13/H3C2/H3C15/H3C12/H3C7 |
| REACTOME_NON_INTEGRIN_MEMBRANE_ECM_INTERACTIONS | REACTOME_NON_INTEGRIN_MEMBRANE_ECM_INTERACTIONS | 48 | 0.639206 | 1.758975 | 0.000118 | 0.002217 | 0.001829 | 891 | tags=50%, list=17%, signal=42% | COL11A1/COL1A1/COL1A2/COL3A1/COL2A1/FN1/LAMA4/COL4A1/COL5A2/COL4A2/LAMB1/LAMA5/ITGB4/SNTG2/AGRN/LAMA1/SNTA1/DDR1/COL5A1/LAMC3/SDC2/LAMC2/SDC3/PDGFB |
| REACTOME_HDMS_DEMETHYLATE_HISTONES | REACTOME_HDMS_DEMETHYLATE_HISTONES | 19 | -0.69436 | -2.40382 | 0.000165 | 0.002906 | 0.002397 | 880 | tags=74%, list=17%, signal=61% | KDM6A/H4C8/KDM7A/H4C6/H4C4/H4C3/H4C13/H3C3/H3C11/H3C8/H3C2/H3C15/H3C12/H3C7 |
| REACTOME_G2_M_DNA_DAMAGE_CHECKPOINT | REACTOME_G2_M_DNA_DAMAGE_CHECKPOINT | 31 | -0.56714 | -2.01216 | 0.000166 | 0.002906 | 0.002397 | 741 | tags=55%, list=14%, signal=47% | DNA2/H4C8/H4C6/RMI1/H2BC5/BLM/CCNB1/H4C4/H2BC9/H2BC17/H4C3/H4C13/H2BC6/H2BC3/CCNA2/H2BC13/CDK1 |
| REACTOME_SIGNALING_BY_RECEPTOR_TYROSINE_KINASES | REACTOME_SIGNALING_BY_RECEPTOR_TYROSINE_KINASES | 203 | 0.494196 | 1.486103 | 0.000164 | 0.002906 | 0.002397 | 1269 | tags=37%, list=24%, signal=29% | PGR/COL11A1/COL1A1/PTN/COL1A2/APOE/COL3A1/IGF2/EGFR/PGF/MET/SHC2/COL2A1/FN1/NTRK3/COL6A1/FLRT2/FGFR3/CDH5/LAMA4/GABRB2/COL4A1/COL5A2/PTPRU/YAP1/COL4A2/LAMB1/THBS2/LAMA5/COL6A2/BCAR1/LAMA1/COL9A3/NTRK2/COL5A1/ADAM12/F3/NRG3/LAMC3/ID3/COL6A3/ID1/FAM83B/SPHK1/PDGFRB/PTPRF/COL9A1/LAMC2/FGFR1/ID4/FLRT1/PDGFB/SHB/SPP1/MAPK11/GRB7/FGFRL1/ESRP1/FGF18/AXL/BAIAP2/MDK/GIPC1/PTPRS/SPRED1/FGF10/DOCK3/VEGFB/ERBB2/SPARC/PIK3R2/DLG4/ERBB3/VEGFA/PLAT |
| REACTOME_HATS_ACETYLATE_HISTONES | REACTOME_HATS_ACETYLATE_HISTONES | 51 | -0.5041 | -1.93325 | 0.000173 | 0.002959 | 0.002441 | 1155 | tags=61%, list=22%, signal=48% | ENY2/H2BC21/SUPT20H/TADA1/DR1/KAT2B/ELP4/ING3/KAT6B/H4C8/H4C6/H2AC12/H2BC5/H2AC16/H4C4/H2AC14/H2BC9/H2BC17/H2AC13/H4C3/H4C13/H2BC6/H3C3/H3C11/H2BC3/H3C8/H2BC13/H3C2/H3C15/H3C12/H3C7 |
| REACTOME_TRANSPORT_OF_BILE_SALTS_AND_ORGANIC_ACIDS_METAL_IONS_AND_AMINE_COMPOUNDS | REACTOME_TRANSPORT_OF_BILE_SALTS_AND_ORGANIC_ACIDS_METAL_IONS_AND_AMINE_COMPOUNDS | 20 | 0.737722 | 1.804078 | 0.000181 | 0.003027 | 0.002497 | 415 | tags=40%, list=8%, signal=37% | SLC6A1/SLC13A4/SLC6A13/SLC47A1/SLC6A20/SLC22A8/SLC22A3/SLC13A3 |
| REACTOME_RUNX1_REGULATES_GENES_INVOLVED_IN_MEGAKARYOCYTE_DIFFERENTIATION_AND_PLATELET_FUNCTION | REACTOME_RUNX1_REGULATES_GENES_INVOLVED_IN_MEGAKARYOCYTE_DIFFERENTIATION_AND_PLATELET_FUNCTION | 37 | -0.56376 | -2.04264 | 0.000216 | 0.003525 | 0.002908 | 518 | tags=51%, list=10%, signal=47% | H4C6/H2BC5/H4C4/H2AC14/H2BC9/GATA1/H2BC17/H4C3/H4C13/H2BC6/H3C3/H3C11/H2BC3/H3C8/H2BC13/H3C2/H3C15/H3C12/H3C7 |
| REACTOME_RUNX1_REGULATES_TRANSCRIPTION_OF_GENES_INVOLVED_IN_DIFFERENTIATION_OF_HSCS | REACTOME_RUNX1_REGULATES_TRANSCRIPTION_OF_GENES_INVOLVED_IN_DIFFERENTIATION_OF_HSCS | 37 | -0.55515 | -2.01144 | 0.00029 | 0.004631 | 0.00382 | 518 | tags=54%, list=10%, signal=49% | H4C6/H2BC5/H4C4/H2AC14/H2BC9/GATA1/H2BC17/H4C3/H4C13/H2BC6/H3C3/MYB/H3C11/H2BC3/H3C8/H2BC13/H3C2/H3C15/H3C12/H3C7 |
| REACTOME_REGULATION_OF_ENDOGENOUS_RETROELEMENTS_BY_PIWI_INTERACTING_RNAS_PIRNAS | REACTOME_REGULATION_OF_ENDOGENOUS_RETROELEMENTS_BY_PIWI_INTERACTING_RNAS_PIRNAS | 38 | -0.53512 | -1.96332 | 0.000307 | 0.004802 | 0.003961 | 518 | tags=50%, list=10%, signal=45% | H4C6/H2BC5/H4C4/H2AC14/H2BC9/PIWIL4/H2BC17/H4C3/H4C13/H2BC6/H3C3/H3C11/H2BC3/H3C8/H2BC13/H3C2/H3C15/H3C12/H3C7 |
| REACTOME_CHROMOSOME_MAINTENANCE | REACTOME_CHROMOSOME_MAINTENANCE | 41 | -0.51414 | -1.90814 | 0.000331 | 0.004971 | 0.0041 | 741 | tags=54%, list=14%, signal=46% | DNA2/H4C8/CENPL/CENPU/H4C6/H2BC5/BLM/H4C4/H2AC14/H2BC9/H2BC17/KNL1/H4C3/H4C13/H2BC6/H2BC3/CCNA2/HJURP/H2BC13/CENPA/CENPI/OIP5 |
| REACTOME_DISEASES_OF_METABOLISM | REACTOME_DISEASES_OF_METABOLISM | 101 | 0.530859 | 1.557919 | 0.000329 | 0.004971 | 0.0041 | 1159 | tags=40%, list=22%, signal=31% | BGN/THSD4/SI/ADAMTSL1/NOTCH3/PRELP/FMOD/MUC3A/BCAN/MUC17/THBS2/GPC1/MUC4/LUM/AGRN/SEMA5A/ADAMTS2/MUC5AC/MUC6/GPC6/ADAMTS7/PC/GPC4/SDC2/ACAN/ADAMTSL5/ADAMTSL3/CSPG4/ADAMTSL2/SDC3/CYP26B1/SEMA5B/ADAMTS12/ABCA3/DCN/SBSPON/ADAMTS10/ADAMTS9/HSPG2/B4GAT1 |
| REACTOME_DIFFERENTIATION_OF_KERATINOCYTES_IN_INTERFOLLICULAR_EPIDERMIS_IN_MAMMALIAN_SKIN | REACTOME_DIFFERENTIATION_OF_KERATINOCYTES_IN_INTERFOLLICULAR_EPIDERMIS_IN_MAMMALIAN_SKIN | 15 | 0.76985 | 1.800461 | 0.000358 | 0.005269 | 0.004347 | 934 | tags=80%, list=18%, signal=66% | SPRR2A/YAP1/KRT14/KRT19/ITGB4/DMKN/KLK7/ABCG2/CSPG4/SPINK5/TP63/KRT5 |
| REACTOME_REPLACEMENT_OF_PROTAMINES_BY_NUCLEOSOMES_IN_THE_MALE_PRONUCLEUS | REACTOME_REPLACEMENT_OF_PROTAMINES_BY_NUCLEOSOMES_IN_THE_MALE_PRONUCLEUS | 14 | -0.71895 | -2.20803 | 0.000395 | 0.005696 | 0.004699 | 738 | tags=79%, list=14%, signal=68% | H4C8/H4C6/H2BC5/H4C4/H2BC9/H2BC17/H4C3/H4C13/H2BC6/H2BC3/H2BC13 |
| REACTOME_RECOGNITION_AND_ASSOCIATION_OF_DNA_GLYCOSYLASE_WITH_SITE_CONTAINING_AN_AFFECTED_PURINE | REACTOME_RECOGNITION_AND_ASSOCIATION_OF_DNA_GLYCOSYLASE_WITH_SITE_CONTAINING_AN_AFFECTED_PURINE | 16 | -0.69229 | -2.17986 | 0.000405 | 0.005721 | 0.00472 | 738 | tags=88%, list=14%, signal=75% | H2BC21/POT1/H4C8/H4C6/H2BC5/H4C4/H2AC14/H2BC9/H2BC17/H4C3/H4C13/H2BC6/H2BC3/H2BC13 |
| REACTOME_MITOTIC_PROPHASE | REACTOME_MITOTIC_PROPHASE | 50 | -0.47449 | -1.83575 | 0.000482 | 0.006685 | 0.005515 | 518 | tags=44%, list=10%, signal=40% | H4C6/H2BC5/CCNB1/H4C4/H2AC14/H2BC9/PLK1/H2BC17/H4C3/H4C13/H2BC6/H3C3/H3C11/H2BC3/H3C8/H2BC13/H3C2/H3C15/H3C12/H3C7/CDK1/CCNB2 |
| REACTOME_DISEASES_OF_IMMUNE_SYSTEM | REACTOME_DISEASES_OF_IMMUNE_SYSTEM | 10 | -0.76904 | -2.2039 | 0.000515 | 0.006762 | 0.005578 | 926 | tags=90%, list=18%, signal=74% | IRAK4/CHUK/BTK/TLR3/CD36/TLR1/FGB/FGA/FGG |
| REACTOME_RNA_POLYMERASE_I_PROMOTER_ESCAPE | REACTOME_RNA_POLYMERASE_I_PROMOTER_ESCAPE | 33 | -0.54655 | -1.96754 | 0.000505 | 0.006762 | 0.005578 | 518 | tags=55%, list=10%, signal=49% | H4C6/H2BC5/H4C4/H2AC14/H2BC9/H2BC17/H4C3/H4C13/H2BC6/H3C3/H3C11/H2BC3/H3C8/H2BC13/H3C2/H3C15/H3C12/H3C7 |
| REACTOME_NERVOUS_SYSTEM_DEVELOPMENT | REACTOME_NERVOUS_SYSTEM_DEVELOPMENT | 203 | 0.483805 | 1.454856 | 0.000508 | 0.006762 | 0.005578 | 887 | tags=30%, list=17%, signal=26% | GAP43/MYH14/COL3A1/EGFR/EFNB3/MET/COL2A1/DPYSL5/DSCAM/NTN1/COL6A1/UNC5C/SLIT3/COL4A1/ITGA10/EPHA5/COL5A2/DOK5/YAP1/COL4A2/LAMB1/COL6A2/GPC1/ARHGAP39/SPTBN4/ENAH/AGRN/SCN4B/DPYSL3/LAMA1/SEMA5A/COL9A3/KCNQ3/COL5A1/CACNA1C/TUBB3/MSI1/WWTR1/EFNA2/CACNA1H/TUBB2B/EFNB2/TEAD1/COL6A3/SDC2/CRMP1/SCN2B/PLXNB1/PRX/TREM2/RGMA/ALCAM/COL9A1/UNC5B/TUBB6/FGFR1/PAK4/SLIT2/SCN5A/CLTB |
| REACTOME_KIDNEY_DEVELOPMENT | REACTOME_KIDNEY_DEVELOPMENT | 17 | 0.744755 | 1.792244 | 0.000537 | 0.006919 | 0.005707 | 865 | tags=71%, list=17%, signal=59% | FOXC2/SIX2/FOXC1/SIX1/OSR1/EYA1/PCDH19/NPNT/HNF4A/BMP4/ID4/SLIT2 |
| REACTOME_SENESCENCE_ASSOCIATED_SECRETORY_PHENOTYPE_SASP | REACTOME_SENESCENCE_ASSOCIATED_SECRETORY_PHENOTYPE_SASP | 35 | -0.53355 | -1.89955 | 0.000582 | 0.007374 | 0.006083 | 518 | tags=54%, list=10%, signal=49% | H4C6/H2BC5/H4C4/H2AC14/H2BC9/H2BC17/H4C3/H4C13/H2BC6/H3C3/H3C11/H2BC3/H3C8/CCNA2/H2BC13/H3C2/H3C15/H3C12/H3C7 |
| REACTOME_DEVELOPMENTAL_LINEAGE_OF_PANCREATIC_ACINAR_CELLS | REACTOME_DEVELOPMENTAL_LINEAGE_OF_PANCREATIC_ACINAR_CELLS | 13 | -0.71445 | -2.18591 | 0.000621 | 0.007733 | 0.006379 | 89 | tags=54%, list=2%, signal=53% | PRSS1/PNLIPRP2/CPB1/CELA3B/CPA1/CPA2/PNLIP |
| REACTOME_INTEGRIN_CELL_SURFACE_INTERACTIONS | REACTOME_INTEGRIN_CELL_SURFACE_INTERACTIONS | 47 | 0.610322 | 1.677534 | 0.000704 | 0.008626 | 0.007115 | 1175 | tags=68%, list=23%, signal=53% | COL1A1/COL1A2/COL16A1/COL3A1/COL2A1/COL8A1/JAM2/FN1/COL6A1/COL4A1/ITGA10/COL5A2/COL4A2/COL6A2/LUM/ITGA11/AGRN/COL9A3/COL5A1/COL7A1/COL6A3/COL8A2/COL9A1/COL23A1/SPP1/COL10A1/COL18A1/ITGA7/ITGB8/HSPG2/ICAM1/CDH1 |
| REACTOME_PROCESSING_OF_DNA_DOUBLE_STRAND_BREAK_ENDS | REACTOME_PROCESSING_OF_DNA_DOUBLE_STRAND_BREAK_ENDS | 31 | -0.53356 | -1.893 | 0.000724 | 0.008724 | 0.007196 | 741 | tags=52%, list=14%, signal=45% | DNA2/H4C8/H4C6/RMI1/H2BC5/BLM/H4C4/H2BC9/H2BC17/H4C3/H4C13/H2BC6/H2BC3/CCNA2/H2BC13/CLSPN |
| REACTOME_DISEASES_ASSOCIATED_WITH_O_GLYCOSYLATION_OF_PROTEINS | REACTOME_DISEASES_ASSOCIATED_WITH_O_GLYCOSYLATION_OF_PROTEINS | 35 | 0.635971 | 1.679199 | 0.000792 | 0.009388 | 0.007744 | 1234 | tags=63%, list=24%, signal=48% | THSD4/ADAMTSL1/NOTCH3/MUC3A/MUC17/THBS2/MUC4/SEMA5A/ADAMTS2/MUC5AC/MUC6/ADAMTS7/ADAMTSL5/ADAMTSL3/ADAMTSL2/SEMA5B/ADAMTS12/SBSPON/ADAMTS10/ADAMTS9/B4GAT1/ADAMTS4 |
| REACTOME_CYCLIN_A_B1_B2_ASSOCIATED_EVENTS_DURING_G2_M_TRANSITION | REACTOME_CYCLIN_A_B1_B2_ASSOCIATED_EVENTS_DURING_G2_M_TRANSITION | 14 | -0.7009 | -2.15258 | 0.0009 | 0.010503 | 0.008664 | 461 | tags=64%, list=9%, signal=59% | CCNB1/PLK1/CCNA2/CDC25A/HJURP/PKMYT1/CDK1/CCNB2/TICRR |
| REACTOME_HDACS_DEACETYLATE_HISTONES | REACTOME_HDACS_DEACETYLATE_HISTONES | 36 | -0.53909 | -1.94015 | 0.000944 | 0.010838 | 0.00894 | 518 | tags=58%, list=10%, signal=53% | H4C6/H2AC12/H2BC5/H2AC16/H4C4/H2AC14/H2BC9/H2BC17/H2AC13/H4C3/H4C13/H2BC6/H3C3/H3C11/H2BC3/H3C8/H2BC13/H3C2/H3C15/H3C12/H3C7 |
| REACTOME_SYNDECAN_INTERACTIONS | REACTOME_SYNDECAN_INTERACTIONS | 16 | 0.734735 | 1.747567 | 0.000959 | 0.010841 | 0.008943 | 845 | tags=56%, list=16%, signal=47% | COL1A1/COL1A2/COL3A1/FN1/COL5A2/ITGB4/COL5A1/SDC2/SDC3 |
| REACTOME_MITOTIC_SPINDLE_CHECKPOINT | REACTOME_MITOTIC_SPINDLE_CHECKPOINT | 37 | -0.52256 | -1.89336 | 0.001005 | 0.011188 | 0.009229 | 673 | tags=46%, list=13%, signal=40% | CENPL/CENPU/NDC80/CENPE/PLK1/KNL1/BUB1B/ERCC6L/BUB1/CDCA8/KIF2C/CENPF/CENPA/AURKB/CENPI/SKA1/BIRC5 |
| REACTOME_NCAM1_INTERACTIONS | REACTOME_NCAM1_INTERACTIONS | 21 | 0.704848 | 1.739121 | 0.001144 | 0.012549 | 0.010352 | 760 | tags=67%, list=15%, signal=57% | COL3A1/COL2A1/COL6A1/COL4A1/COL5A2/COL4A2/COL6A2/AGRN/COL9A3/COL5A1/CACNA1C/CACNA1H/COL6A3/COL9A1 |
| REACTOME_OXIDATIVE_STRESS_INDUCED_SENESCENCE | REACTOME_OXIDATIVE_STRESS_INDUCED_SENESCENCE | 41 | -0.48015 | -1.78198 | 0.001284 | 0.013879 | 0.011449 | 518 | tags=44%, list=10%, signal=40% | H4C6/H2BC5/H4C4/H2AC14/H2BC9/H2BC17/H4C3/H4C13/H2BC6/H3C3/H3C11/H2BC3/H3C8/H2BC13/H3C2/H3C15/H3C12/H3C7 |
| REACTOME_BASE_EXCISION_REPAIR_AP_SITE_FORMATION | REACTOME_BASE_EXCISION_REPAIR_AP_SITE_FORMATION | 17 | -0.64153 | -2.05364 | 0.00146 | 0.014907 | 0.012297 | 738 | tags=82%, list=14%, signal=71% | H2BC21/POT1/H4C8/H4C6/H2BC5/H4C4/H2AC14/H2BC9/H2BC17/H4C3/H4C13/H2BC6/H2BC3/H2BC13 |
| REACTOME_REGULATION_OF_ENDOGENOUS_RETROELEMENTS_BY_KRAB_ZFP_PROTEINS | REACTOME_REGULATION_OF_ENDOGENOUS_RETROELEMENTS_BY_KRAB_ZFP_PROTEINS | 34 | -0.52943 | -1.87214 | 0.001446 | 0.014907 | 0.012297 | 518 | tags=53%, list=10%, signal=48% | H4C6/H2BC5/H4C4/H2AC14/H2BC9/H2BC17/H4C3/H4C13/H2BC6/H3C3/H3C11/H2BC3/H3C8/H2BC13/H3C2/H3C15/H3C12/H3C7 |
| REACTOME_HOMOLOGY_DIRECTED_REPAIR | REACTOME_HOMOLOGY_DIRECTED_REPAIR | 41 | -0.47507 | -1.76313 | 0.00143 | 0.014907 | 0.012297 | 518 | tags=46%, list=10%, signal=42% | DNA2/H4C8/H4C6/RMI1/H2BC5/BLM/H4C4/POLE2/H2BC9/H2BC17/H4C3/H4C13/H2BC6/H2BC3/CCNA2/RAD51/H2BC13/POLQ/CLSPN |
| REACTOME_SIGNALING_BY_PDGF | REACTOME_SIGNALING_BY_PDGF | 34 | 0.633061 | 1.667608 | 0.001438 | 0.014907 | 0.012297 | 971 | tags=50%, list=19%, signal=41% | COL3A1/COL2A1/COL6A1/COL4A1/COL5A2/COL4A2/THBS2/COL6A2/BCAR1/COL9A3/COL5A1/COL6A3/PDGFRB/COL9A1/PDGFB/SPP1/GRB7 |
| REACTOME_REGULATION_OF_INSULIN_LIKE_GROWTH_FACTOR_IGF_TRANSPORT_AND_UPTAKE_BY_INSULIN_LIKE_GROWTH_FACTOR_BINDING_PROTEINS_IGFBPS | REACTOME_REGULATION_OF_INSULIN_LIKE_GROWTH_FACTOR_IGF_TRANSPORT_AND_UPTAKE_BY_INSULIN_LIKE_GROWTH_FACTOR_BINDING_PROTEINS_IGFBPS | 54 | 0.57256 | 1.598976 | 0.00172 | 0.017315 | 0.014283 | 985 | tags=54%, list=19%, signal=44% | ITIH2/MXRA8/APOE/IGF2/CCN1/IGFBP6/IGFBP5/FN1/PENK/LAMB1/GAS6/FAM20A/C4A/IGFBP4/FSTL3/VWA1/IGFBP2/KLK1/SDC2/IGFBP7/SERPIND1/BMP4/PCSK9/APP/FAM20C/SPP1/MMP2/STC2/SPARCL1 |
| REACTOME_FORMATION_OF_THE_CORNIFIED_ENVELOPE | REACTOME_FORMATION_OF_THE_CORNIFIED_ENVELOPE | 23 | 0.674361 | 1.691278 | 0.001797 | 0.017615 | 0.014531 | 1352 | tags=78%, list=26%, signal=58% | KRT4/DSP/SPRR2A/SPRR1B/KRT14/KRT19/KRT6C/KRT6B/KRT6A/SPINK5/PKP3/PKP2/PKP1/KRT5/PPL/PRSS8/DSG2/KRT18 |
| REACTOME_KERATINIZATION | REACTOME_KERATINIZATION | 23 | 0.674361 | 1.691278 | 0.001797 | 0.017615 | 0.014531 | 1352 | tags=78%, list=26%, signal=58% | KRT4/DSP/SPRR2A/SPRR1B/KRT14/KRT19/KRT6C/KRT6B/KRT6A/SPINK5/PKP3/PKP2/PKP1/KRT5/PPL/PRSS8/DSG2/KRT18 |
| REACTOME_HEDGEHOG_ON_STATE | REACTOME_HEDGEHOG_ON_STATE | 29 | 0.647627 | 1.669673 | 0.001907 | 0.018445 | 0.015216 | 335 | tags=28%, list=6%, signal=26% | BOC/HHIP/GAS1/GLI2/DZIP1/GLI1/GPR161/SMO |
| REACTOME_NCAM_SIGNALING_FOR_NEURITE_OUT_GROWTH | REACTOME_NCAM_SIGNALING_FOR_NEURITE_OUT_GROWTH | 34 | 0.626234 | 1.649625 | 0.001934 | 0.01846 | 0.015228 | 809 | tags=47%, list=16%, signal=40% | COL3A1/COL2A1/COL6A1/COL4A1/COL5A2/COL4A2/COL6A2/SPTBN4/AGRN/COL9A3/COL5A1/CACNA1C/CACNA1H/COL6A3/COL9A1/FGFR1 |
| REACTOME_NEGATIVE_EPIGENETIC_REGULATION_OF_RRNA_EXPRESSION | REACTOME_NEGATIVE_EPIGENETIC_REGULATION_OF_RRNA_EXPRESSION | 37 | -0.50267 | -1.82129 | 0.002286 | 0.021543 | 0.017772 | 518 | tags=49%, list=10%, signal=44% | H4C6/H2BC5/H4C4/H2AC14/H2BC9/H2BC17/H4C3/H4C13/H2BC6/H3C3/H3C11/H2BC3/H3C8/H2BC13/H3C2/H3C15/H3C12/H3C7 |
| REACTOME_AMYLOID_FIBER_FORMATION | REACTOME_AMYLOID_FIBER_FORMATION | 36 | -0.50582 | -1.82041 | 0.002383 | 0.022171 | 0.018289 | 518 | tags=58%, list=10%, signal=53% | H4C6/H2BC5/H4C4/H2AC14/H2BC9/H2BC17/H4C3/H4C13/H2BC6/H3C3/H3C11/H2BC3/H3C8/H2BC13/H3C2/H3C15/H3C12/H3C7/TTR/LTF/FGA |
| REACTOME_B_WICH_COMPLEX_POSITIVELY_REGULATES_RRNA_EXPRESSION | REACTOME_B_WICH_COMPLEX_POSITIVELY_REGULATES_RRNA_EXPRESSION | 35 | -0.49266 | -1.75397 | 0.002448 | 0.02249 | 0.018552 | 518 | tags=51%, list=10%, signal=47% | H4C6/H2BC5/H4C4/H2AC14/H2BC9/H2BC17/H4C3/H4C13/H2BC6/H3C3/H3C11/H2BC3/H3C8/H2BC13/H3C2/H3C15/H3C12/H3C7 |
| REACTOME_DNA_DAMAGE_TELOMERE_STRESS_INDUCED_SENESCENCE | REACTOME_DNA_DAMAGE_TELOMERE_STRESS_INDUCED_SENESCENCE | 23 | -0.57578 | -1.98553 | 0.002498 | 0.022667 | 0.018698 | 518 | tags=61%, list=10%, signal=55% | H4C6/H2BC5/H4C4/H2AC14/H2BC9/H2BC17/CCNE2/H4C3/H4C13/H2BC6/H2BC3/CCNA2/H1-5/H2BC13 |
| REACTOME_FORMATION_OF_THE_URETERIC_BUD | REACTOME_FORMATION_OF_THE_URETERIC_BUD | 10 | 0.772627 | 1.668606 | 0.002962 | 0.026549 | 0.021901 | 865 | tags=80%, list=17%, signal=67% | FOXC2/SIX2/FOXC1/SIX1/EYA1/NPNT/BMP4/SLIT2 |
| REACTOME_BASE_EXCISION_REPAIR | REACTOME_BASE_EXCISION_REPAIR | 22 | -0.56225 | -1.91206 | 0.003019 | 0.026737 | 0.022056 | 518 | tags=59%, list=10%, signal=53% | H4C8/H4C6/H2BC5/H4C4/H2AC14/POLE2/H2BC9/H2BC17/H4C3/H4C13/H2BC6/H2BC3/H2BC13 |
| REACTOME_LAMININ_INTERACTIONS | REACTOME_LAMININ_INTERACTIONS | 19 | 0.686456 | 1.669728 | 0.003877 | 0.033926 | 0.027986 | 1142 | tags=74%, list=22%, signal=58% | LAMA4/COL4A1/COL4A2/LAMB1/LAMA5/ITGB4/LAMA1/NID2/COL7A1/LAMC3/LAMC2/COL18A1/ITGA7/HSPG2 |
| REACTOME_NEUROTRANSMITTER_RELEASE_CYCLE | REACTOME_NEUROTRANSMITTER_RELEASE_CYCLE | 10 | 0.763759 | 1.649454 | 0.003957 | 0.034214 | 0.028224 | 722 | tags=60%, list=14%, signal=52% | SLC6A1/SLC6A13/CPLX1/SYN1/STXBP1/PPFIA3 |
| REACTOME_RESOLUTION_OF_SISTER_CHROMATID_COHESION | REACTOME_RESOLUTION_OF_SISTER_CHROMATID_COHESION | 46 | -0.42048 | -1.58714 | 0.004693 | 0.039649 | 0.032707 | 673 | tags=46%, list=13%, signal=40% | CENPL/CENPU/NDC80/CENPE/CCNB1/PLK1/KNL1/BUB1B/ERCC6L/BUB1/CDCA8/KIF2C/CENPF/CENPA/AURKB/CENPI/CDCA5/CDK1/CCNB2/SKA1/BIRC5 |
| REACTOME_SIGNALING_BY_GPCR | REACTOME_SIGNALING_BY_GPCR | 159 | 0.462449 | 1.382133 | 0.004652 | 0.039649 | 0.032707 | 1034 | tags=35%, list=20%, signal=29% | EGFR/WNT6/RGS11/GAL/FN1/CX3CL1/PENK/ADCY5/GNG13/FZD7/SSTR2/FGD1/WNT5A/EDNRA/GCGR/MMP3/SMO/WNT9A/DGKB/ARHGEF25/SAA1/CAMK2B/ARHGEF16/OPRD1/GALR1/ADRB1/PPP1R1B/GNG12/DRD4/ARHGEF17/GNA11/PDE8B/PLXNB1/PTGDR/ARHGEF10/APLNR/GPSM1/ADRA1B/ADCY6/APLN/PLEKHG5/HTR1D/ARHGEF26/RAMP2/FZD2/APP/FZD8/CCR10/PDE10A/FZD1/WNT2/ARHGEF4/GPR4/AGT/HCAR1/ARHGEF38 |
| REACTOME_MATERNAL_TO_ZYGOTIC_TRANSITION_MZT | REACTOME_MATERNAL_TO_ZYGOTIC_TRANSITION_MZT | 36 | -0.47909 | -1.72421 | 0.005181 | 0.042783 | 0.035293 | 518 | tags=50%, list=10%, signal=45% | H4C6/H2BC5/H4C4/H2AC14/H2BC9/H2BC17/H4C3/H4C13/H2BC6/H3C3/H3C11/H2BC3/H3C8/H2BC13/H3C2/H3C15/H3C12/H3C7 |
| REACTOME_DISEASES_OF_PROGRAMMED_CELL_DEATH | REACTOME_DISEASES_OF_PROGRAMMED_CELL_DEATH | 37 | -0.47423 | -1.71826 | 0.005132 | 0.042783 | 0.035293 | 518 | tags=51%, list=10%, signal=47% | H4C6/H2BC5/H4C4/H2AC14/H2BC9/H2BC17/H4C3/H4C13/H2BC6/H3C3/H3C11/H2BC3/H3C8/CDC25A/H2BC13/H3C2/H3C15/H3C12/H3C7 |
| REACTOME_REPRODUCTION | REACTOME_REPRODUCTION | 39 | -0.46578 | -1.68012 | 0.005436 | 0.044392 | 0.03662 | 518 | tags=54%, list=10%, signal=49% | H4C6/H2BC5/CATSPERB/BLM/H4C4/H2AC14/H2BC9/H2BC17/H4C3/H4C13/H2BC6/H3C3/H3C11/H2BC3/H3C8/RAD51/H2BC13/H3C2/H3C15/H3C12/H3C7 |
| REACTOME_DISEASES_ASSOCIATED_WITH_GLYCOSAMINOGLYCAN_METABOLISM | REACTOME_DISEASES_ASSOCIATED_WITH_GLYCOSAMINOGLYCAN_METABOLISM | 24 | 0.648323 | 1.643136 | 0.005534 | 0.044694 | 0.036869 | 1000 | tags=58%, list=19%, signal=47% | BGN/PRELP/FMOD/BCAN/GPC1/LUM/AGRN/GPC6/GPC4/SDC2/ACAN/CSPG4/SDC3/DCN |
| REACTOME_O_GLYCOSYLATION_OF_TSR_DOMAIN_CONTAINING_PROTEINS | REACTOME_O_GLYCOSYLATION_OF_TSR_DOMAIN_CONTAINING_PROTEINS | 19 | 0.674822 | 1.641432 | 0.006089 | 0.04865 | 0.040132 | 1234 | tags=79%, list=24%, signal=60% | THSD4/ADAMTSL1/THBS2/SEMA5A/ADAMTS2/ADAMTS7/ADAMTSL5/ADAMTSL3/ADAMTSL2/SEMA5B/ADAMTS12/SBSPON/ADAMTS10/ADAMTS9/ADAMTS4 |
| REACTOME_INTERLEUKIN_7_SIGNALING | REACTOME_INTERLEUKIN_7_SIGNALING | 12 | -0.66036 | -1.96178 | 0.006277 | 0.049605 | 0.04092 | 544 | tags=75%, list=10%, signal=67% | HGF/H3C3/H3C11/H3C8/H3C2/RAG1/H3C15/H3C12/H3C7 |
