# Supplementary material for: Analysis of the Matrix Metalloproteinases Family Profile in Gastric Cancer Suggests Key Matrix Metalloproteinases for Tumor Development and Their Clinical Impact
Source: Mol Carcinog. 2026 Feb 23;65(5):577–88. doi: 10.1002/mc.70097 (PMC13067799; doi:10.1002/mc.70097)
Supplement: Supplementary file 8 — Supporting Material Table 7 ‐ Analysis of the expression of housekeeping genes in samples from patients with gastric cancer. [file MC-65-577-s005.docx]

**Supplementary Material Table 7 -  Analysis of the expression of housekeeping genes in samples from patients with gastric cancer.**

| **Gene** | **n** | **mean_vst** | **sd_vst** | **cv** |
| --- | --- | --- | --- | --- |
| EEF1A1 | 342 | 13.52511088 | 0.682580606 | 0.050468 |
| ACTB | 342 | 12.97292241 | 0.840884538 | 0.064818 |
| SDHA | 342 | 7.980218963 | 0.600270279 | 0.07522 |
| RPLP0 | 342 | 10.46607746 | 0.822020366 | 0.078541 |
| PPIA | 342 | 9.82697292 | 0.785601703 | 0.079943 |
| TBP | 342 | 5.816676156 | 0.472765802 | 0.081278 |
| GAPDH | 342 | 11.05481834 | 0.903907754 | 0.081766 |
| RPL13A | 342 | 10.91579128 | 1.069672266 | 0.097993 |
| HPRT1 | 342 | 5.976992112 | 0.632372134 | 0.105801 |
| B2M | 342 | 12.55845404 | 1.529939377 | 0.121825 |
