# Supplementary material for: Analysis of the Matrix Metalloproteinases Family Profile in Gastric Cancer Suggests Key Matrix Metalloproteinases for Tumor Development and Their Clinical Impact
Source: Mol Carcinog. 2026 Feb 23;65(5):577–88. doi: 10.1002/mc.70097 (PMC13067799; doi:10.1002/mc.70097)
Supplement: Supplementary file 9 — Supporting Material Table 8 ‐ Analysis of the expression of housekeeping genes in gastric cancer samples, with distribution in tumoral (CG) and peritumoral (PTT) regions. [file MC-65-577-s006.docx]

**Supplementary Material Table 8 -  Analysis of the expression of housekeeping genes in gastric cancer samples, , with distribution in tumoral (CG) and peritumoral (PTT) regions.**

| **Gene** | **n_GAC** | **n_PTT** | **delta_median_GAC_minus_PTT** | **wilcox_p** | **comparison** | **wilcox_q** |
| --- | --- | --- | --- | --- | --- | --- |
| B2M | 156 | 186 | -0.271981214 | 0.000896722 | GAC vs PTT | 0.0089672 |
| GAPDH | 156 | 186 | 0.266087104 | 0.005495836 | GAC vs PTT | 0.0274792 |
| ACTB | 156 | 186 | 0.145618916 | 0.069933262 | GAC vs PTT | 0.2331109 |
| EEF1A1 | 156 | 186 | -0.115037797 | 0.196404322 | GAC vs PTT | 0.4910108 |
| TBP | 156 | 186 | 0.027968953 | 0.247352299 | GAC vs PTT | 0.4947046 |
| RPLP0 | 156 | 186 | 0.070126993 | 0.353196492 | GAC vs PTT | 0.5045664 |
| HPRT1 | 156 | 186 | -0.044404626 | 0.331979256 | GAC vs PTT | 0.5045664 |
| PPIA | 156 | 186 | 0.051833335 | 0.820619139 | GAC vs PTT | 0.8206191 |
| RPL13A | 156 | 186 | 0.037306174 | 0.800189328 | GAC vs PTT | 0.8206191 |
| SDHA | 156 | 186 | 0.031535321 | 0.800613596 | GAC vs PTT | 0.8206191 |
